# Structures of flavivirus RNA promoters suggest two binding modes with NS5 polymerase

**DOI:** 10.1038/s41467-021-22846-1

**Published:** 2021-05-05

**Authors:** Eunhye Lee, Paul J. Bujalowski, Tadahisa Teramoto, Keerthi Gottipati, Seth D. Scott, Radhakrishnan Padmanabhan, Kyung H. Choi

**Affiliations:** 1 Department of Biochemistry and Molecular Biology, Sealy Center for Structural Biology and Molecular Biophysics, The University of Texas Medical Branch, 301 University Boulevard, Galveston, TX USA; 2 Department of Microbiology and Immunology, Georgetown University School of Medicine, Washington, DC USA

**Keywords:** RNA, Dengue virus, X-ray crystallography

## Abstract

Flaviviruses use a ~70 nucleotide stem-loop structure called stem-loop A (SLA) at the 5′ end of the RNA genome as a promoter for RNA synthesis. Flaviviral polymerase NS5 specifically recognizes SLA to initiate RNA synthesis and methylate the 5′ guanosine cap. We report the crystal structures of dengue (DENV) and Zika virus (ZIKV) SLAs. DENV and ZIKV SLAs differ in the relative orientations of their top stem-loop helices to bottom stems, but both form an intermolecular three-way junction with a neighboring SLA molecule. To understand how NS5 engages SLA, we determined the SLA-binding site on NS5 and modeled the NS5-SLA complex of DENV and ZIKV. Our results show that the gross conformational differences seen in DENV and ZIKV SLAs can be compensated by the differences in the domain arrangements in DENV and ZIKV NS5s. We describe two binding modes of SLA and NS5 and propose an SLA-mediated RNA synthesis mechanism.

## Abstract (web summary)

Flaviviruses use a ~70 nucleotide stem-loop structure called stem-loop A (SLA) at the 5’ end of the RNA genome as a promoter for RNA synthesis by the viral polymerase NS5. Here the authors describe the structures of dengue and Zika virus SLAs, identify the SLA-binding site on NS5, and propose models for how NS5 recognizes the RNA promoter.

## Introduction

Flaviviruses such as dengue (DEN), West Nile (WN), Japanese encephalitis (JE) and Zika virus (ZIKV), cause viral hemorrhagic fever and/or encephalitis in humans. For example, dengue virus (DENV) causes diseases ranging in severity from mild dengue fever with non-specific flu-like symptoms to fatal dengue hemorrhagic fever and dengue shock syndrome^1^. The incidence of DENV infection has increased dramatically in recent decades and the CDC estimates 400 million dengue infections occur each year^2^. Similarly, recent outbreaks of ZIKV in the Americas show that the virus is linked to microcephaly in newborns and Guillain-Barre syndrome in adults^3^. Despite the significant impact of flavivirus infection on human health, vaccines are available only for a limited number of flaviviruses, and antiviral therapies to treat viral infections are not available. Thus, there is pressing need to develop additional therapeutics and vaccines that interfere with essential steps in the flaviviral lifecycle.

Flaviviruses are enveloped viruses that encapsidate a positive-sense RNA genome of ~11 kb, which consists of a 5’-cap, a 5’ untranslated region (5’-UTR), a single open-reading frame (ORF), and a 3’-UTR. The ORF is translated as a single polyprotein, which is later processed into three structural proteins (capsid (C), pre-membrane (prM), and envelope (E)), and seven non-structural (NS) proteins (1, 2A, 2B, 3, 4A, 4B, and 5). The viral NS proteins, along with viral RNA and unidentified host proteins, self-assemble on the cytoplasmic side of the ER membrane to form a viral replication complex that carries out genome replication^4^. Among the NS proteins, NS3 and NS5 enzymatic activities are required for genome replication. NS3 consists of an N-terminal serine protease, which requires NS2B as a cofactor, and a C-terminal helicase. The helicase domain also has 5’-RNA triphosphatase activity (which hydrolyzes the γ-phosphate of RNA for capping)^5–7^. NS5, the largest NS protein (103 kDa), consists of an N-terminal methyltransferase (MTase) domain and a C-terminal RNA-dependent RNA polymerase (RdRp) domain. The MTase domain has guanylyltransferase and methyltransferase activities and is involved in capping and methylation to form a type 1 cap structure at the 5’-end of the genome^8–10^. The 5′- and 3′-UTRs have conserved RNA secondary structures that are essential for viral replication. In particular, the 5’-UTR includes a ~70 nucleotide long stem-loop, called stem-loop A (SLA) that functions as a promoter for viral RNA synthesis by NS5 polymerase^11–14^. NS5 recognizes and interacts with SLA at least twice during genome replication. NS5 first recognizes the SLA to initiate negative-strand RNA synthesis at the 3’ end of the genome (RdRp function)^11,15,16^. Later, during positive-strand RNA synthesis, NS5 again recognizes SLA and methylates the 5’ guanine cap to form an ^m7^GpppA^m^ type 1 cap (MTase function)^17–19^.

SLA-mediated RNA replication is likely conserved in flaviviruses. Flavivirus SLAs share a similar, predicted ‘Y’-shaped 3-way junction structure despite sharing only low sequence identity^20^. Flavivirus NS5 from one species can bind an SLA from a related species with similar affinities as for its cognate SLA, and successfully replicate the genome of the related species^21,22^. For example, chimeric WNV containing DENV serotype 2 (DENV2) SLA at its 5’-end is able to replicate similarly to wild-type WNV^21^. Despite the essential role SLA plays in flavivirus genome replication, little is known regarding the structure of SLA or how NS5 recognizes and engages SLA for replication or methylation. Here we report crystal structures of flavivirus SLA; DENV and ZIKV SLA were determined to 3.4 and 3.8 Å resolution, respectively. Both structures show similar structural elements, consisting of a top stem-loop, a side loop, and a bottom stem, but differ in size and the relative arrangements of the individual elements. We identified the SLA-binding sites in their respective NS5 viral polymerases and show that the structural differences in DENV and ZIKV SLAs correlate with the differences in the domain arrangements of their NS5s.

## Results

### Structures of flavivirus SLAs were determined using a tRNA scaffold

The SLA structures were determined using a chimeric RNA, wherein the DENV2 SLA (nucleotides 1–70) or ZIKV SLA (nucleotides 1–71) was inserted into the anticodon loop of human tRNA^Lys^ (tRNA-SLA^DENV^ and tRNA-SLA^ZIKV^, Supplementary Fig. 1)^23^. The linker region between the tRNA and SLA was modified to form a dsRNA helix to provide stability to the structure. The tRNA scaffold strategy was based on the known propensity of tRNAs to maintain the structure and function of inserted RNAs and to form diffraction quality crystals^23^. The tRNA scaffold was indeed essential for crystallization, since the tRNA moiety was involved in crystal contacts in both the DENV and ZIKV SLA structures (Supplementary Fig. 2). Thus, tRNA scaffolds could be used as part of a generalized approach for the crystallization of large RNA molecules. The tRNA-SLA structures were determined by molecular replacement using yeast tRNA^Phe^ and short dsRNA helices as search models. For both DENV and ZIKV SLA, the entire tRNA-SLA^DENV^ (139 nt) and for tRNA-SLA^ZIKV^ (142 nt) structures could be traced in the electron density maps. Data collection and refinement statistics for DENV and ZIKV SLA are shown in Supplementary Table 1.

**Fig. 1:**
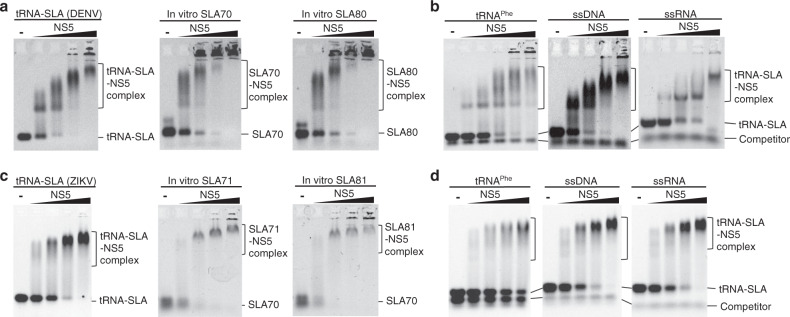
NS5 binding analysis of tRNA-fused stem-loop A (tRNA-SLA). **a** Interaction between DENV NS5 and SLA. Interactions between NS5 and various SLAs (tRNA-SLA^DENV^ and in vitro transcribed SLA_70_ and SLA_80_) were examined by electrophoretic mobility shift assay (EMSA). SLA_70_ and SLA_80_ contain the first 70 and 80 nucleotides of DENV2 RNA genome, respectively. The tRNA-SLA^DENV^, SLA_70_ and SLA_80_ (1 μM) were titrated with increasing concentrations of NS5 from 1 μM to 4 μM. NS5 binds tRNA-SLA, SLA_70_, and SLA_80_ similarly. EMSA was performed twice with similar results. **b** Competition assay between tRNA-SLA^DENV^ and other nucleic acids for DENV NS5. The tRNA-SLA^DENV^ (1 μM) was titrated with increasing concentrations of DENV NS5 in the presence of tRNA^phe^, ssDNA (43 nt), and ssRNA (8 nt) (1 μM each). The nucleic acids were stained with ethidium bromide and visualized under UV at 302 nm. The competition assay was repeated twice with similar results. **c** Interaction between ZIKV NS5 and SLA. Interactions between NS5 and various SLAs (tRNA-SLA^ZIKV^ and in vitro transcribed SLA_71_ and SLA_81_) were analyzed by EMSA. EMSA was carried out similarly as in **a**. EMSA was performed twice with similar results. **d** Competition assay between tRNA-SLA^ZIKV^ and other nucleic acids for ZIKV NS5. Competition assay with tRNA^phe^, ssDNA, and ssRNA was carried out as described above, and repeated twice with similar results. Source data for figures a-d are provided as a Source Data file.

To test if SLA fused with the tRNA-scaffold maintains its fold, we measured the binding constant between DENV NS5 and tRNA-SLA^DENV^. The tRNA-SLA^DENV^ binds viral polymerase NS5 with binding constant of 4.8 × 10^6^ M^−1^ using a fluorescence competition assay (Supplementary Fig. 3). This value is similar to the binding constants determined for the unmodified DENV SLAs (7.0 ×10^6^−1.0 ×10^7^ M^−1^)^22^. To further test if tRNA-SLA constructs form similar NS5 complexes as the unmodified SLA, interactions of tRNA-SLA^DENV^ and tRNA-SLA^ZIKV^ with their respective NS5s were analyzed using electrophoretic mobility shift assays (EMSA) (Fig. 1). The tRNA-SLA^DENV^ and tRNA-SLA^ZIKV^ bind their respective NS5s similarly as in vitro transcribed SLA (i.e. without the tRNA scaffold), indicating that SLA retains its native fold in the tRNA-SLA chimera (Fig. 1a, c). The EMSA analysis of SLA and NS5 shows multiple bands for RNA-protein complexes, similar to those observed for DENV NS5 and 5’ UTR^24^. Flavivirus NS5 is likely in a monomer-dimer equilibrium in solution. The NS5 dimer was detected by size exclusion chromatography with multiangle light scattering (SEC-MAL), analytical ultracentrifugation, and X-ray crystallography^25–28^. We found that SLA also forms a dimer in solution (see below), and thus, the multiple bands in EMSA may represent NS5 monomer/dimer and SLA monomer/dimer complexes. We next tested if NS5 and tRNA-SLA interactions are specific using a competition assay with different forms of nucleic acids, such as yeast tRNA^Phe^, single-stranded DNA (ssDNA), and single-stranded RNA (ssRNA). DENV NS5 specifically binds to tRNA-SLA^DENV^ in the presence of yeast tRNA^Phe^, ssDNA or ssRNA (Fig. 1b). The interaction between ZIKV NS5 and tRNA-SLA^ZIKV^ was less specific than that of DENV. Although ZIKV NS5 specifically binds to tRNA-SLA^ZIKV^ in the presence of ssDNA or ssRNA, the protein showed slightly reduced binding in the presence of tRNA^Phe^ (Fig. 1d).

### DENV SLA is a large ‘L’-shaped molecule and forms a dimer via kissing loop interactions

**Fig. 2:**
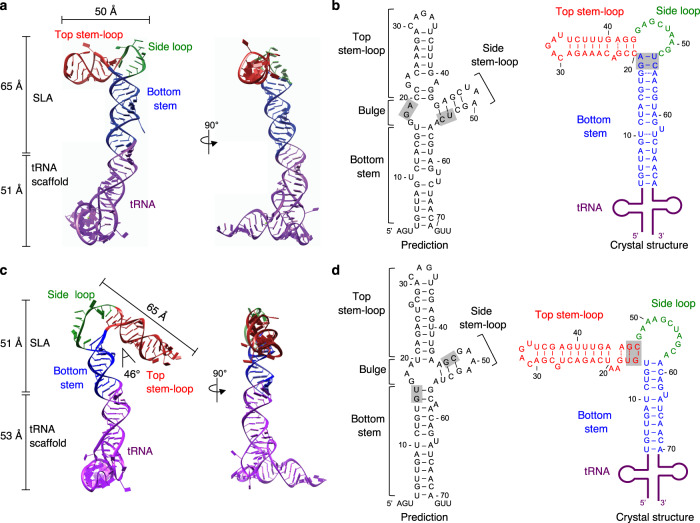
Structure of flavivirus stem-loop A (SLA). **a** Structure of DENV SLA. The structure of tRNA-SLA^DENV^ is shown in cartoon models with top stem-loop (red), side loop (green), and bottom stem (blue). The tRNA scaffold is shown in purple. **b** Comparison of the predicted and determined secondary structures of DENV SLA. The predicted secondary structures of DENV and ZIKV SLA were generated with the RNAfold program^53^. The secondary structure based on the crystal structure is colored as in (**a**). The shaded areas in the prediction form base pairs in the crystal structure, and thus the predicted side stem-loop does not form. Instead, the side loop becomes single stranded (see main text). **c** Structure of ZIKV SLA. The structure of tRNA-SLA^ZIKV^ is shown in cartoon models with top stem-loop (red), side loop (green), bottom stem (blue), and tRNA scaffold (purple). **d** Comparison of the predicted and determined secondary structures of ZIKV SLA. The shaded areas in the prediction form base pairs in the crystal structure, and the predicted side stem-loop becomes single-stranded side loop.

**Fig. 3:**
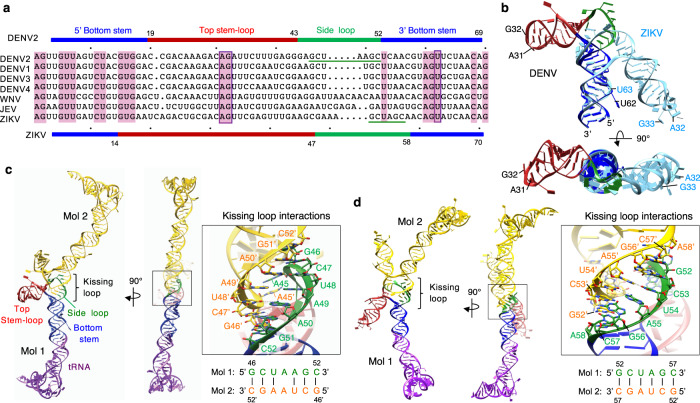
Comparison of DENV and ZIKV stem-loop A (SLA) structures. **a** Sequence alignment of flavivirus SLAs. The SLA sequences from DENV1 (NCBI accession number, NC_001477), DENV2 (NC_001474), DENV3 (NC_001475), DENV4 (NC_002640), WNV (NC_001563), JEV (NC_001437) and ZIKV (KU527068) are aligned. The overall sequence identity among the seven listed SLAs is ~25%. Conserved nucleotides are shaded in pink. The top stem-loop, side loop, and the bottom stem are indicated for DENV2 (top) and ZIKV SLA (bottom). The AG motif and the U-bulge are boxed, and the side loop regions in DENV and ZIKV SLAs involved in the kissing-loop interaction are underlined. **b** Superposition of the DENV and ZIKV SLA structures. The DENV and ZIKV SLA structures were overlaid by the conserved bottom stem. DENV SLA is colored as in Fig. 2a and ZIKV SLA in light blue. The nucleotides in the AG motif and the U-bulge are indicated in DENV (black letters) and ZIKV (blue letters). The top stem-loops of DENV and ZIKV SLAs are related by ~180° rotation. **c** Kissing-loop interactions in the DENV SLA dimer. One tRNA-SLA^DENV^ molecule is shown as in Fig. 2a, and the other molecule is shown in yellow. The self-complementary, side loop sequence that forms kissing-loop interactions is shown below. **d** Kissing-loop interactions in the ZIKV SLA dimer. The tRNA-SLA^ZIKV^ dimer is colored as in Fig. 2c. The palindromic sequence that forms kissing loop interactions between the side loops is shown below.

The tRNA-SLA^DENV^ retained the tRNA^Lys^ structure, suggesting that the SLA moiety also maintains its own fold (Fig. 2). DENV SLA is a 65 Å long by 50 Å wide, ‘L’-shaped molecule, consisting of a top stem-loop (9 bp), a side loop (9 nt), and a bottom stem (16 bp) (Fig. 2a). The top stem-loop is roughly perpendicular to the bottom stem, which extends from the tRNA moiety. When compared to the predicted secondary structure, the structure of DENV SLA has two major differences near the critical 3-way junction, where the top stem-loop, side loop and the bottom stem meet (Fig. 2b). First, the predicted bulge (^17^GGA^19^) between the top and bottom stem does not exist. Instead, the ^18^GA^19^ form Watson-Crick base pairs with the ^53^UC^54^ that was predicted to be in the side stem, resulting in the bottom stem being extended by 3 base pairs compared to the prediction. Second, as a consequence of the extension of the bottom stem (i.e., base pairs between ^18^GA^19^ and ^53^UC^54^), the internal base pairs between ^44^GAGC^47^ and ^51^GCUC^54^ in the predicted side stem cannot form. Thus, the predicted side stem becomes a single-stranded side loop. Surprisingly, the side loop reveals the previously undetected self-complementary sequence (^46^GCUAAGC^52^) that forms intermolecular Watson-Crick base pairs with the equivalent region from the second SLA molecule in palindromic order with one mismatch (Fig. 3). The two SLA molecules form a continuous helical stack from the bottom stem of one SLA molecule, through the kissing loop, to the bottom stem of the second SLA molecule. The side loop-side loop helix is further stabilized by π-π stacking interactions between two ^45^A bases in the dimer (see Fig. 3c). As a consequence of these interactions, DENV SLA dimer has a unique intermolecular three-way (3-way) junction structure, where one helix of the 3-way junction is shared between the two SLA molecules.

### ZIKV SLA is a ‘V’-shaped molecule and forms a dimer via kissing loop interactions

The DENV and ZIKV SLAs share ~58% sequence identity and are predicted to have similar ‘Y’-shaped structures (Figs. 2, 3a), but ZIKV SLA shows different orientations of top stem-loop and side loop compared to DENV SLA. The ZIKV SLA is a 51 Å long by 65 Å wide, consisting of a top stem-loop (13 bp), a side loop (11 nt), and a bottom stem (11 bp). The top stem-loop helix is bent toward the bottom stem at a 46° angle, rendering ZIKV SLA a ‘V’-shaped molecule, rather than the ‘L’ shape observed for DENV SLA (Fig. 2c). The predicted secondary structure for ZIKV SLA was also incorrect near the 3-way junction. The top stem-loop is extended by base pairs between ^15^GU^16^ and ^46^GC^47^, which were predicted to be in the bottom stem and the side stem-loop, respectively (Fig. 2d). The bulge (^17^GAA^19^), predicted to be between the top and bottom stem, does not exist, and is inserted in the extended top stem-loop. Because of base pairing between ^15^GU^16^ and ^46^GC^47^, the internal base pairs in the predicted side stem-loop (between ^45^AGC^47^ and ^52^GCU^54^) do not form, and the side loop of ZIKV SLA is single stranded. Surprisingly, the side loop also contains a previously unidentified, palindromic sequence (^52^GCUAGC^57^) that forms intermolecular Watson-Crick base pairs with equivalent region from the neighboring molecule (see Fig. 3d). This side loop-side loop interaction in the ZIKV SLA dimer results in a dsRNA helix that is coaxially stacked, similar to the DENV SLA dimer. Hence, ZIKV SLA dimer also forms an unusual intermolecular 3-way junction structure where one helix is shared between two SLA molecules instead of the predicted intramolecular 3-way junction.

The DENV and ZIKV SLA structures are consistent with previous selective 2′-hydroxyl acylation analyzed by primer extension (SHAPE)^29,30^. SHAPE suggests whether a nucleotide is flexible or involved in interactions with another nucleotide, i.e., more flexible conformations have a greater probability of having higher SHAPE reactivity. The predicted bulges in DENV (^17^GGA^19^) and ZIKV SLA (^17^GAA^19^) show a relatively low SHAPE reactivity, as the bulges are incorporated into the stems and constrained by base pairing and stacking interactions (Supplementary Fig. 4). The low SHAPE reactivity of the side loop is also expected from its kissing-loop interactions with another side loop because SHAPE cannot distinguish intramolecular vs. intermolecular interactions (Supplementary Fig. 4).

### DENV and ZIKV SLAs have different relative orientations of top and bottom stem, but share conserved features required for viral replication

The DENV and ZIKV SLAs have different tertiary structures in terms of the length of the individual RNA elements (top stem-loop, side loop, and bottom stem) and the relative orientations of the top and bottom stems (Fig. 3b). When the DENV and ZIKV SLA structures are superposed by their conserved bottom stems, the top stem-loop helix and side loop of ZIKV SLA is related to the equivalent regions in DENV SLA by an approximately 180° rotation (Fig. 3b). This difference in orientation occurs because the bottom stem in ZIKV SLA is shorter than that in DENV SLA by 5 base pairs (16 bp vs. 11 bp), which corresponds to ~half-a-turn of a dsRNA helix, leading to an ~180° rotation of the top stem-loop. The top stem-loop in ZIKV SLA is 4 bp longer than that of DENV SLA, leading to an additional approximate half-turn of the top stem helix. The side loop in ZIKV SLA is also 2 nt longer than that of DENV SLA, making the top stem-loop in ZIKV SLA angled towards the bottom stem.

Flavivirus NS5s are shown to use SLAs from related flaviviruses for viral replication despite low sequence identity between the SLAs (Fig. 3a)^21,31^. Thus, NS5 likely recognizes the overall shape and common structural features of SLAs rather than specific sequences. We analyzed functionally important regions in the SLA structures. First, the bottom stem is the most conserved region in SLAs and contains the absolutely conserved ‘U-bulge’ that consists of at least one unpaired U (Fig. 3a). Deletion or mutation of the U-bulge (^62^U) in DENV SLA was shown to be lethal for viral replication, but did not affect affinity for NS5 or in vitro RNA synthesis^32^. In both DENV and ZIKV SLA structures, the bottom stem forms a long dsRNA helix with an unpaired U-bulge located in the same position relative to the 5’ terminus (Fig. 3b). The U-bulge (^62^U) in DENV SLA is flipped out from the dsRNA helix, while the U-bulge (^63^U) in ZIKV SLA is not. It has been shown that base pairing patterns within the bottom stem, rather than the presence of specific bases, are important for viral replication^12,14,31^. Thus, the base pairing in the bottom stem helix likely provides structural stability and helps orient the U-bulge in relation to the 5’ terminus of SLA for interaction with other components of the replication complex.

Second, the top stem-loop in SLA contains a conserved ‘AG’ motif, the only nucleotides conserved outside the bottom stem (Fig. 3a). Substitution or deletion of the AG loop or a shortening of the top stem in flavivirus SLAs completely abolished viral replication^14,31^, suggesting that the position of the AG motif within SLA is important for replication. The top loop (^30^CAGA^33^) in DENV SLA was also identified as the NS5 interaction site in a DENV SLA-NS5 foot-printing experiment^32^. Although the top stem-loops in DENV and ZIKV SLA are related by 180° rotation, the unpaired AG motif is facing the same side because the top stem loop in ZIKV SLA is longer by an additional half turn (Fig. 3b). This suggests that the same face of DENV and ZIKV SLAs, which presents the AG motif, interacts with NS5 (see below).

Last, the side loop of SLA does not have any sequence homology among flaviviruses and is variable in length (Fig. 3a). Tick-borne flaviviruses may have >40 nt long side stems^31^. However, despite the lack of sequence conservation, deletion of the side loop abolishes DENV replication, indicating that the presence of the side loop is essential for viral replication^31^. The SLA structures show that the side loop would be absolutely required to maintain the overall fold of SLA and to orient the top stem-loop relative to the bottom stem. Furthermore, many flavivirus SLAs have a self-complementary sequence in the side loop. The side loops of DENV and ZIKV SLA, containing 6-7 nt of self-complementary sequences, mediate kissing loop interactions with a neighboring SLA molecule (Fig. 3c, d).

### The side loop in SLA mediates RNA-RNA interactions in the SLA dimer

**Fig. 4:**
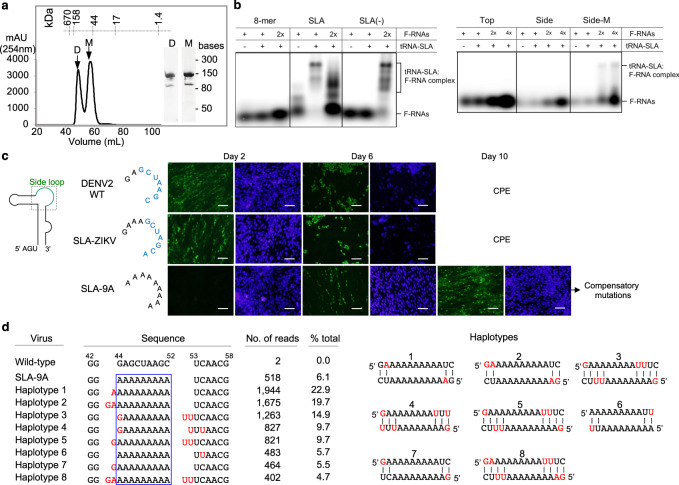
Self-complementarity of the side loop is required for viral replication. **a** The size exclusion chromatography of tRNA-SLA^DENV^. The RNA elutes in two peaks, the early peak likely corresponding to a dimer (D, 91 kDa), and the second peak corresponding to the monomer (M, 45.5 kDa). Protein molecular weight standards are shown on top of the elution profile of tRNA-SLA^DENV^. The size exclusion chromatography was performed twice with similar results. **b** Side loop in SLA mediates RNA-RNA interactions in solution. Interaction between tRNA-SLA^DENV^ and fluorescein-labeled RNAs (F-RNA) was examined by EMSA. See Supplementary Fig. 5 for the RNA sequences. The tRNA-SLA^DENV^ interacts with SLA, SLA(-), and the ssRNA complementary to the side loop sequence (Side-M). The tRNA-SLA^DENV^ does not bind 8-mer random sequence (negative control), the ssRNA complementary to the top stem (Top), or the self-complementary side-loop RNA (Side). Thus, side loop in SLA mediates RNA-RNA interactions. Binding experiments repeated twice with similar results. Source data are provided as a Source Data file. **c** Replication of DENV containing SLA mutations. DENV2 SLA was replaced with either ZIKV (SLA-ZIKV) or 9 A (SLA-9A) in the side-loop, and replication of WT and mutant viruses were monitored by immunofluorescence staining using monoclonal anti-NS1 antibody (green fluorescence color) and nuclear staining with 4’,6-diamidino-2-phenylindole (DAPI, blue fluorescent color). Scale bar represents 50 μm. Replication experiments were repeated three times with similar results. **d** RNA-seq analysis of recovered SLA-9A virus. The numbers and percentage of each haplotypes were calculated from 8,489 total reads. The cutoff of inclusion is >4% abundance. Altered nucleotides in 8 haplotypes are shown in red. Potential intermolecular side-loop interactions resulting from the mutations are shown on the right.

Because both DENV and ZIKV SLAs crystallized as a dimer, we tested if SLA forms a dimer in solution. The tRNA-SLA^DENV^ construct elutes in two peaks in size-exclusion column, suggesting that SLA exists in monomer-dimer equilibrium (Fig. 4a). We also used EMSA and tested if tRNA-SLA^DENV^ can interact with another SLA molecule or the complementary sequence of SLA at the 3’-end of the negative strand, referred to as SLA(-) (Fig. 4b). If SLA(-) has a similar structure as SLA, the side loop sequence of SLA(-) would be complementary to the side loop sequence of SLA. The SLA, SLA(-), or ssRNA were synthesized with a fluorescein label at the 5’-end (Supplementary Fig. 5). The tRNA-SLA^DENV^ interacts with both SLA and SLA(-), indicating that DENV SLA can form both an SLA homodimer and an SLA-SLA(-) heterodimer. A random 8-mer RNA (negative control) does not bind SLA, indicating that the RNA-RNA interactions involving SLA are specific.

We next tested if formation of the SLA homodimer or heterodimer is mediated by the side loop of SLA. Single-stranded RNAs complementary to the top stem and side loop sequences (ssRNA_Top_ and ssRNA_Side_, respectively) were designed with a fluorescein tag at the 5’-end (Supplementary Fig. 5). Due to its self-complementary sequence, the ssRNA_Side_ likely forms a dimer, preventing interaction with tRNA-SLA^DENV^. We thus designed an additional RNA, ssRNA_Side-M_, which contains the side loop sequence and an 8-mer (GC)_4_ extension (Supplementary Fig. 5). The ssRNA_Side-M_ would form a homodimer via the GC extension, allowing the side loop sequence to interact with SLA. As expected, ssRNA_Side-M_ interacts with SLA, while ssRNA_Side_ does not, indicating that the side loop of SLA is single-stranded in solution and mediates specific RNA-RNA interactions (Fig. 4b). The ssRNA_Top_ also does not interact with tRNA-SLA^DENV^, likely because the top stem is already engaged in base pairing and thus unavailable for interaction with SLA (Fig. 4b). Thus, the SLA homodimer and heterodimer interactions are mediated by the side loop, consistent with the SLA structure.

### Self-complementarity of the side loop is important for viral replication

The kissing loop interactions observed in both DENV and ZIKV SLAs, and the conservation of similar self-complementary sequences in other flaviviruses suggest that RNA-RNA interactions via the side loop in SLA may have biological significance. As precedence, intermolecular RNA-RNA interactions in viral genomes of retrovirus and hepatitis C virus have been reported as essential features for viral replication and genome packaging^33–35^. To determine whether the self-complementarity of the side loop sequence plays a role in viral replication, we replaced either the entire DENV SLA (1-70 nt) with that of ZIKV (SLA-ZIKV) or the side loop sequence of DENV SLA (^44^GAGCUAAGC^52^) with ^44^AAAAAAAA^52^ (SLA-9A) in the full-length DENV2 infectious RNA. The SLA-ZIKV mutant would maintain dimer formation, while the SLA-9A mutant would not allow dimer formation. After viral RNAs were transfected into baby hamster kidney cells BHK-21, viral NS1 protein expression and virus production were monitored. WT DENV2 RNA and the SLA-ZIKV mutant showed immunofluorescence-positive cells on day 2 post-transfection and cytopathic effect (CPE) on day 6 (Fig. 4c). In contrast, the SLA-9A mutant was replication defective and did not show any detectable immunofluorescence-positive cells until day 6 post-transfection (Fig. 4c). The cells did not show any CPE even after 10 days post-transfection. To determine whether adaptive mutations were acquired in SLA-9A, viral RNA extracted at day 14 from the supernatant was analyzed using the Sanger sequencing. The recovered SLA-9A virus showed mixed population around the replaced 9 A region. Thus, the viral populations were further analyzed by the RNA-seq method. Eight major haplotypes were observed in the replaced side loop region (Fig. 4d). They have either an insertion of G, A or GA at position 44 (the first nucleotide of the SLA-9A sequence), an insertion of U or UU at position 53 (following the last nucleotide in the SLA-9A sequence), or both (Fig. 4d). These ^44^GA^45^ or ^53^UU^54^ insertions would allow base pairing with ^53^UC^54^ or ^44^AA^45^, respectively, and partially restore the self-complementarity in the side loop. These results indicate that the self-complementarity in the side loop, and not the specific nucleotide sequence, is required for efficient viral replication. In comparison, the WT or SLA-ZIKV virus did not have any significant (>3% frequency) mutations in the entire genome.

### SLA binds NS5 via the methyltransferase and the thumb subdomain of the polymerase domain

**Fig. 5:**
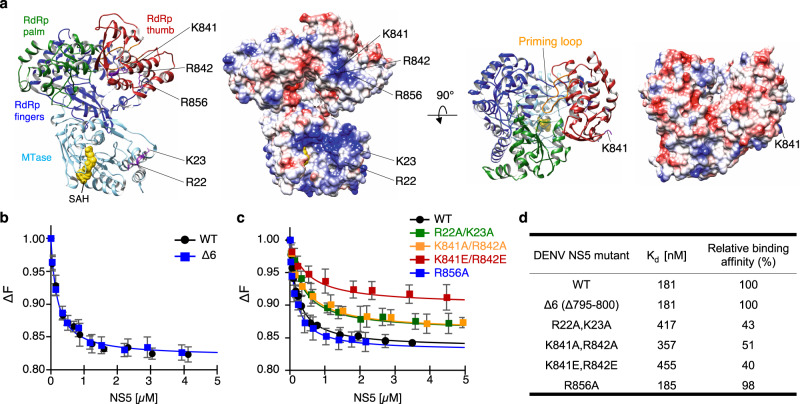
Identification of the stem-loop A (SLA)-binding site on DENV NS5. **a** Structure of DENV NS5. The locations of the residues selected for mutational analysis are indicated in the ribbon diagram and electrostatic surface of DENV NS5 (PDB 4V0Q. DENV NS5 is colored by its domains, the MTase (cyan) and RdRp (fingers, blue; palm, green; thumb, red). The S-adenosyl homocysteine (SAH, yellow) bound in the MTase active site and the priming loop in the thumb subdomain (orange) are indicated. **b** Interaction between NS5-Δ6 and SLA. The binding affinities of DENV SLA with wild-type NS5 and the priming-loop deletion mutant NS5-Δ6 were determined by fluorescence titration using fluorescein-labeled SLA. The NS5-Δ6 binds SLA with the wild-type affinity (181 nM), and thus SLA does not bind in the template-binding channel. The assays were performed in triplicate (n=3 independent experiments). Error bars represent the standard deviations. **c** Identification of the SLA-binding site on DENV NS5. Positively charged residues in the MTase and RdRp domains were mutated and their interactions with SLA were measured using fluorescein-labeled SLA. The assays were performed in triplicate (n=3 independent experiments). Error bars represent the standard deviations. **d** The dissociation constants and relative binding affinities of NS5 mutants. The apparent dissociation constants (*K*_*d*_) calculated from the fluorescence titration curves (**b** and **c**) are listed.

Flavivirus replication is initiated when the viral polymerase NS5 identifies the viral genome via specific recognition of the SLA^12,16,32^. Accordingly, flavivirus NS5 binds the SLA promoter with high affinity and high selectivity, yet how NS5 specifically engages SLA is not clear^12,36^ (Fig. 1). To identify the SLA-binding site in NS5, we first tested if SLA binds in the template-binding channel of NS5. Flavivirus NS5 consists of MTase and RdRp domains, and initiates RNA synthesis via a de novo mechanism (i.e. without a primer). The flavivirus RdRps use a large thumb subdomain and a priming loop that encircles the active site to restrict the volume of the template-binding channel, allowing only ssRNA template to enter the active site during de novo initiation^28,37^. SLA is mostly composed of dsRNA helices that are too large to fit into the template-binding channel without the conformational changes of the priming loop in the thumb subdomain (Fig. 5a). Since deletion of a priming loop in the RdRp has been shown to result in an NS5 capable of accommodating a large dsRNA^28,38^, we measured the interaction between fluorescein-labeled DENV SLA and an NS5 mutant NS5-Δ6, in which six amino acids (^795^WSIHAH^800^) in the priming loop of the thumb subdomain had been deleted. The priming loop deletion mutant (NS5-Δ6) binds SLA with a wild-type affinity of 181 nM, indicating that SLA does not bind in the template-binding channel (Fig. 5b). Therefore, NS5 must have two distinct RNA-binding sites: the template-binding channel for ssRNA template, and the SLA-binding site.

We next examined the size and charge complementarity of NS5 and SLA structures^28,39–42^. The large size of SLA (65 ×50 Å) suggests that the complementary SLA-binding surface on NS5 is similarly large. The full-length DENV NS5 is ~90 ×70 Å in size^28^, slightly larger than SLA. Thus, both RdRp and MTase domains of NS5 likely interact with SLA. Functionally, both NS5 domains are required to interact with SLA, i.e., the MTase for 5’-cap methylation and the RdRp for viral genome replication^11,15,16,43^. We identified positively charged MTase and RdRp surfaces on the same face of NS5 that likely bind the negatively charged SLA, R22 and K23 in the MTase and K841, R842, and R856 in the thumb subdomain of RdRp (Fig. 5a and Supplementary Fig. 6a). We mutated the residues and measured the interactions with fluorescein-labeled SLA (Fig. 5c). The R22A/K23A, K841A/R842A, and K841E/R842E mutants show a decreased dissociation constant (*K*_*d*_) of 417 nM (43% of WT), 357 nM (51% of WT) and 455 nM (40% of WT), respectively, indicating that the SLA-binding site in DENV NS5 involves R22-K23 and K841-R842 (Fig. 5c). In contrast, R856A has wild-type binding affinity, suggesting that R856 is not involved in SLA interaction (Fig. 5c). Interestingly, mutations in the identified SLA-binding site have been shown to reduce viral replication. A R22-K23 mutation in DENV4 NS5 reduces viral replication by 1,000 fold, and a K841-R842 mutation in DENV2 NS5 abolishes viral replication^44–46^. Since these residues are not directly involved in catalysis of MTase and RdRp, they may be defective in viral replication due to reduced SLA binding.

**Fig. 6:**
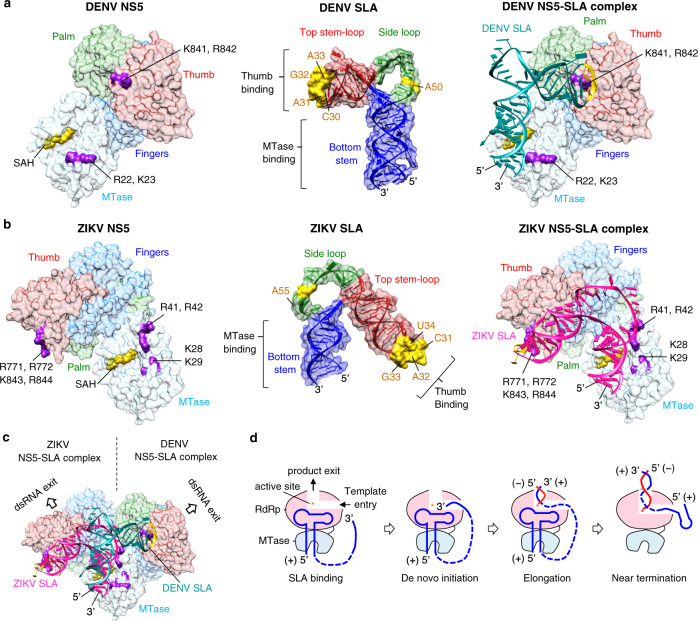
Model of the flavivirus NS5 and stem-loop A (SLA) complexes. **a** The DENV NS5-SLA complex. DENV NS5 (left), SLA (middle), and its interaction (right) are shown. DENV NS5 surface (PDB 4V0Q) is colored by the domains, the MTase (cyan) and RdRp (fingers, blue; palm, green; thumb, red), and the SLA-binding site residues are colored in purple. The S-adenosyl homocysteine (SAH) bound in the MTase active site is depicted in yellow. DENV SLA is colored by its region (top stem-loop, red; side loop, green; bottom stem, blue) and the nucleotides implicated in NS5 interactions^32^ are colored in yellow. The NS5-SLA complex is modeled by placing the 5’ terminus of SLA near the MTase active site, and the top stem-loop and side loop of SLA toward the RdRp domain. **b** The ZIKV NS5-SLA complex. ZIKV NS5 surface (PDB code 5U0B) is colored by its domains as in **a** with the SLA-binding site^47^ colored in purple (left). ZIKV SLA structure is colored as in **a** with the nucleotides implicated in DENV NS5-SLA interactions^32^ in yellow (middle). The ZIKV NS5-SLA complex is modeled similarly to the DENV NS5-SLA complex, by placing the 5’ terminus of SLA near the MTase active site, and the top stem-loop and side loop of SLA toward the RdRp domain (right). **c** Superposition of the DENV and ZIKV complexes. The DENV and ZIKV NS5-SLA complexes are overlaid by the MTase that interacts with the SLA bottom stem. The relative arrangement of MTase and RdRp domains in DENV and ZIKV NS5 correlates with the conformations of SLAs. **d** Model for SLA-dependent RNA synthesis. Viral polymerase NS5 binds SLA at the 5’-end of the genome and forms the NS5-SLA complex. The top stem-loop and side loop of SLA interact with the RdRp domain (pink), and the 5’ terminus and the bottom stem of SLA interact with the MTase domain (cyan). Both 5’ SLA and the 3’-end of the viral genome bind simultaneously to NS5 in the SLA-binding site and the template-binding channel, respectively. De novo RNA synthesis begins in the NS5 active site and continues until the entire genome is synthesized. The 5’-end SLA remains bound to NS5 during elongation until it is used as the template near the completion of RNA synthesis. The positive-strand RNA template (+) and negative-strand RNA product (−) are shown in blue and red, respectively.

We have recently determined that ZIKV SLA interacts with the individual MTase and RdRp domains of NS5, and the SLA-binding site in ZIKV NS5 comprises K28-K29-R41-R42 (MTase) and R771-R772-R775-K843-K844 (thumb subdomain of RdRp) (Supplementary Fig. 6b)^47^. Amino acid residues K843-K844 in ZIKV NS5 correspond to K841-R842 in DENV NS5, indicating that similar residues participate in SLA interactions in both DENV and ZIKV NS5. Interestingly, these equivalent residues in DENV3 and ZIKV NS5 are located ~80 Å away from each other when the MTase domains are superposed (see Fig. 6). This suggests that NS5 may have two binding modes for SLA interaction (see next section).

## Discussion

### Modeling of the SLA-NS5 complex suggests two binding modes of flavivirus SLA and NS5

Flavivirus NS5 structures determined thus far show two major relative orientations of the RdRp and MTase domains; DENV3 NS5 adopts one conformation, and ZIKV, YFV and JEV NS5 the other (Supplementary Fig. 7a)^28,39–42,48^. Recently, DENV2 NS5 was crystallized in both conformations^49^, suggesting that the two conformations of NS5 are interconvertible. When the MTase domains of the two conformations are superposed, the RdRp domains are related by a 108° rotation and a ~5 Å translation (Supplementary Fig. 7b). The observed structural differences in NS5 may relate to the conformational differences observed in SLA structures of DENV and ZIKV. Thus, we modeled the DENV and ZIKV NS5-SLA complexes using structural and biochemical data. First, previous DENV NS5-SLA foot-printing assays showed that the ^30^CAGA^33^ nucleotides (the AG motif) in the top stem-loop and ^50^A in the side loop were protected by binding either full-length NS5 or the RdRp domain alone, suggesting that the RdRp domain interacts with the top stem-loop and the side loop (Fig. 6a)^32^. This is also consistent with a report that the K841-R842 residues (thumb subdomain of RdRp) in DENV2 NS5 recognize the ACAG nucleotide in the top stem-loop of SLA^50^. Thus, the top stem-loop and the side loop of SLA were positioned toward the RdRp domain near the positively charged surface involving K841-R842 (DENV3) or K843-K844 (ZIKV) in their respective NS5 (Fig. 6a, b). Second, it was reported that the entire WNV SLA sequence is required for 5’-guanosine monophosphate cap methylation at the N7 position by MTase^43^, suggesting that the 5’-cap at the SLA bottom stem binds to the active site of MTase. Thus, the 5’-terminus of SLA was positioned near the active site of MTase near R22 and K23 (DENV NS5) or K28, K29, R41 and R42 (ZIKV NS5) (Fig. 6a, b). In both DENV and ZIKV NS5-SLA models, the SLAs can be placed across the MTase and RdRp domains without major clashes and interact with their respective NS5s in a similar manner (Fig. 6c). In particular, the rotation of the thumb subdomains in ZIKV NS5 relative to DENV3 NS5 is compensated by the rotation of the top stem-loop in the ZIKV SLA, resulting in the same NS5 residues interacting with the same SLA bases despite the gross conformational differences between DENV and ZIKV SLAs and NS5s. Thus, the arrangement of MTase and RdRp domains in DENV3 and ZIKV NS5 reflects the conformations of their respective SLAs. Thus, flavivirus NS5 and SLA may have coevolved to optimize their interactions during viral replication. If the NS5 conformations are interchangeable as shown for DENV2 NS5^49^, NS5 would be able to bind both DENV and ZIKV SLA structures and replicate similarly. We show that replication of DENV2 RNA containing ZIKV SLA at its 5’ end (SLA-ZIKV in Fig. 4c) was indistinguishable from that of WT DENV2 virus, suggesting that the conformations of DENV2 NS5 enables productive interactions with either form of SLAs.

### The proposed SLA-mediated RNA synthesis mechanism

How NS5 interacts with the SLA structure at the 5’-end of the genome to promote RNA synthesis at the 3’ end is poorly understood. To initiate negative strand RNA synthesis at the 3’-end of the viral genome, the NS5 and SLA complex should recognize and interact with the 3’-end. It is not known whether NS5 can bind SLA and the 3’-end of the viral genome simultaneously during RNA synthesis. Alternatively, SLA and the 3’-end of genome could compete for the template-binding channel in NS5. Our binding assay shows that the deletion of the priming loop of NS5 has no effect on SLA binding (Fig. 5b), indicating that SLA binds in a site different from the template-binding channel. Therefore, SLA and the 3’-end of the genome likely bind NS5 simultaneously to initiate RNA synthesis, explaining how NS5 couples the SLA promoter binding at the 5’-end to RNA synthesis at the 3’-end of genome. Consistent with the binding data, our modeling of the DENV and ZIKV NS5-SLA complexes also suggests that the bound SLA would not obstruct either the template-binding channel or the dsRNA exit site in the RdRp (Fig. 6c). We thus propose an SLA-mediated RNA synthesis mechanism wherein flavivirus NS5 recognizes the large SLA via the high-affinity SLA-binding site, and simultaneously recruits the 3’-end of viral genome near the template-binding channel (Fig. 6d). Then, the 3’-end of the viral genome would be positioned in the polymerase active site to be copied by de novo RNA synthesis^37,51^. Because the SLA-binding site does not overlap with the template-binding channel, SLA would likely stay bound to NS5 during the elongation phase, until it is time for the SLA at the 5’-end to act as a template to finish replication of the end of the genome. This NS5-SLA interaction would provide a simple mechanism for accomplishing three essential tasks during replication: 1) selective synthesis of the viral RNA genome from the myriad of cellular mRNAs in the cytoplasm, 2) sequestering only fully intact viral RNAs on NS5 for replication as opposed to prematurely aborted transcripts, and thus 3) ensuring that a complete copy of the 5’-end of the genome is produced.

## Methods

### Construction, expression, and purification of DENV and ZIKV tRNA-SLA

The tRNA-SLA fusion constructs for DENV2 and ZIKV were designed based on the tRNA scaffold approach^52^. The DENV2 SLA sequence, containing the first 70 nucleotides was inserted into the anticodon loop of human tRNA^Lys^ to generate tRNA-SLA^DENV^. Two additional mutations were introduced to the chimeric tRNA-SLA^DENV^ to stabilize the structure by base pairing in the linker region between the tRNA and SLA moieties. The ^3^U (DENV2 SLA numbering) was replaced with C for base pairing with ^70^G, and two nucleotides CU were inserted after ^70^G to form base pairs with ^1^AG^2^ (Supplementary Fig. 1). The final chimeric tRNA-SLA^DENV^ construct contains 139 nucleotides. Secondary structure prediction using the RNAfold program^53^ indicated that the modified chimeric RNA maintains both the tRNA and SLA structures. DNA encoding the designed tRNA-SLA^DENV^ was synthesized in DNA2.0 (Newark, CA), and inserted into pBluescript II SK vector with *lpp* promoter, the promoter of most abundant *E.coli* lipoprotein, and *E.coli rrnC* terminator. The tRNA-SLA^ZIKV^ was generated from tRNA-SLA^DENV^ by several mutagenesis that replaced the sequence of DENV2 SLA to ZIKV SLA, the first 71 nucleotides of the epidemic strain of ZIKV (KU527068). The chimeric tRNA-SLA^ZIKV^ also contains the modification at ^3^U (ZIKV SLA numbering) to C (to base pair with ^71^G) and the insertion of CU after ^71^G. Because the tRNA-SLA^ZIKV^ construct did not yield diffraction-quality crystals, additional constructs were generated by modifying the linker region between the SLA and the tRNA, from one base pair insertion to 10 base pair deletion by one base pair. The tRNA-SLA^ZIKV^ containing the U-A base-pair insertion in the linker region (142 nt) showed best diffraction and used for structure determination (Supplementary Fig. 1).

The plasmids containing either tRNA-SLA^DENV^ or tRNA-SLA^ZIKV^ were freshly transformed to *E.coli* BL21 cells, and the cells were grown overnight in 2x YT medium at 37 °C. The cultured cells were transferred to 2 liters of 2x YT medium and cultured for 20 h at 37 °C. The cells were harvested and then resuspended in 15 ml of buffer containing 10 mM magnesium acetate and 10 mM Tris-HCl, pH 7.4. The RNA was extracted by adding 10 ml saturated phenol and gently agitating for 1 h at room temperature, and then the solution was centrifuged at 20,198 x g for 30 min at 20 °C to remove cell debris. The aqueous phase was then mixed with 0.1 volume of 5 M NaCl and 2 volumes of absolute ethanol for RNA precipitation. The RNA was pelleted by centrifugation at 20,198 x g for 30 min at 4 °C, and then dissolved in buffer A (40 mM sodium phosphate, pH 7.0) for ion-exchange chromatography. The extracted RNA was loaded on HiLoad 16/60 Q ion-exchange column (GE Healthcare, Buckinghamshire, UK) equilibrated with buffer A. After a washing step with 0.4 M NaCl, RNA was eluted with the 0.4–0.7 M NaCl gradient in buffer A. The fractions were analyzed by electrophoresis on 8 % urea-acrylamide gel. The pooled samples were buffer-exchanged to 20 mM Tris-HCl, pH 7.4, containing 100 mM NaCl and 2 mM MgCl_2_, and concentrated to ~20 mg/ml for crystallization. For size-exclusion chromatography, the RNA was loaded on Superdex S75 HiLoad 16/600 column (GE Healthcare). The tRNA-SLA^DENV^ eluted in two separate peaks that correspond to a dimer and monomer, respectively. The fractions from the two peaks were resolved on 8% urea-PAGE in Tris/borate/EDTA buffer.

### Crystallization, X-ray data collection and structure determination

The tRNA-SLA^DENV^ was crystallized by the sitting-drop vapor diffusion method at 20 °C by mixing the RNA with an equal volume of a reservoir solution containing 100 mM sodium cacodylate, pH 6.9, 9% (w/v) polyethylene glycol 8,000, 200 mM KCl, and 100 mM magnesium acetate. Crystals grew to a full size within a week. For data collection, crystals were transferred to mother liquor supplemented with 20% (v/v) ethylene glycol and flash-frozen in liquid nitrogen. Diffraction data to 3.4 Å resolution were collected at 100 K with a wavelength of 0.9785 Å at the Advanced Photon Source beamline 21-ID-F (Argonne National Laboratory, Chicago). The data set was processed using HKL2000^54^. The crystal belonged to space group I222 with unit cell dimensions of a = 30.9 Å, b = 137.5 Å, c = 336.8 Å, and contained one molecule in the asymmetric unit with solvent content of 69%. The initial solutions were found by molecular replacement with yeast tRNA^Phe^ (PDB 1TN1) and partial RNA helix (PDB 5EW4) as search models using the program PHASER in the PHENIX suite^55^. Manual model building was carried out with Coot and iterative refinement was performed with phenix.refine^55,56^. The final model contained one tRNA-SLA^DENV^ molecule, one magnesium ion, and 7 water molecules with the R and R_free_ factors of 22.6 and 26.8%, respectively (Supplementary Table 1).

The tRNA-SLA^ZIKV^ was crystallized by the sitting-drop vapor diffusion method at 20 °C by mixing the RNA with an equal volume of a reservoir solution containing 100 mM Tris, pH 8.1, 24% (w/v) polyethylene glycol 3350, 200 mM LiSO_4_, and 67 mM CsCl. The crystals grew with salt crystals in the same drop and took 2-3 weeks to reach full size. For X-ray data collection, the crystals were soaked into mother liquor supplemented with 20% (v/v) ethylene glycol for 5-10 min and flash-frozen in liquid nitrogen. Diffraction data to 3.8 Å resolution were collected at 100 K with a wavelength of 1.1271 Å at the Advanced Photon Source beamline 21-ID-D (Argonne National Laboratory, Chicago). The data set was processed with HKL2000^54^ and the crystal belonged to space group C222 with unit cell dimensions of *a* = 157.1 Å, *b* = 156.6 Å, *c* = 72.3 Å. The asymmetric unit contained one molecule with solvent content of 74%. The initial solution was found by molecular replacement with tRNA-SLA^DENV^ as search model using the program PHASER in the PHENIX suite^55^. Extensive model building was required to trace the entire tRNA-SLA^ZIKV^ molecule. Manual model building and iterative refinement were carried out with Coot and phenix.refine^55,56^. The final model contained one tRNA-SLA^ZIKV^ molecule with the R and R_free_ factors of 22.7 and 27.2%, respectively (Supplementary Table 1).

### NS5 and tRNA-SLA interaction by electrophoretic mobility shift assay

DENV NS5 interaction with tRNA-SLA^DENV^ was compared to NS5 interaction with unmodified SLA to determine if the tRNA-SLA maintains the native fold of SLA. The wild-type DENV NS5 was expressed in *E.coli* and purified as previously described^28^. RNAs containing the first 70 and 80 nt of the DENV2 genome, SLA_70_ and SLA_80_, respectively, were prepared by in vitro transcription using the T7 RNA polymerase. Both SLA_70_ and SLA_80_ contain an extra G at the 5’ end to facilitate transcription by T7 RNA polymerase. The DENV NS5 binding activities of tRNA-SLA^DENV^, SLA_70_, and SLA_80_ were observed by EMSA. Briefly, 1 μM of RNA (tRNA-SLA, SLA_70_, or SLA_80_) were incubated with increasing amount of NS5 protein (1 to 4 μM) in 20 μl of binding buffer (20 mM Tris, pH 7.4, 150 mM NaCl, 2 mM MgCl_2_, and 5% glycerol) for 30 min at room temperature. Each reaction mixture was then mixed with 2 μl of 0.01% bromophenol blue dye and resolved on 1% agarose gel in 1x Tris/borate/EDTA (TBE) buffer. ZIKV NS5 interactions with tRNA-SLA^ZIKV^ and in vitro transcribed ZIKV SLAs were similarly determined by EMSA. The ZIKV NS5 was expressed with a SUMO-tag in *E.coli* and purified as previously described^40^. ZIKV RNAs containing the first 71 and 81 nucleotides (SLA_71_ and SLA_81_) were prepared using in vitro RNA transcription.

The specificity of the DENV NS5 and tRNA-SLA^DENV^ interaction was also examined by competitive binding reaction with 1 μM of yeast tRNA^phe^ (Sigma-Aldrich), single-stranded 43mer DNA (5’-CATGTATTTTCACAGAGCGGATCTTCGCCTAGCATCCAACGCC-3’, IDT, Coralville, IA), or fluorescein-labeled 8mer RNA (5’F-AGAAAAGG-3’, IDT, Coralville, IA). The tRNA-SLA^DENV^ and competitor mixture was incubated with increasing amount of DENV NS5 (1-4 μM) for 30 min at room temperature and resolved on 1% agarose gel in 1x TBE buffer. The fluorescein-labeled RNA and other nucleotides including tRNA-SLA^DENV^ were visualized by ethidium bromide staining under UV (302 nm). The specificity of the ZIKV NS5 and tRNA-SLA^ZIKV^ interaction was similarly examined by competitive binding assay with yeast tRNA^phe^, 43mer DNA, and 8-mer RNA.

### RNA-RNA interaction of tRNA-SLA by EMSA

To analyze the intermolecular interaction of tRNA-SLA^DENV^ through the side loop, DENV2 SLA containing the first 80 nucleotides (SLA) and the 3’-end of DENV2 negative strand that is complementary to SLA, SLA(-), were synthesized with fluorescein at the 5’-end by IDT (Coralville, IA) (Supplementary Fig. 5). The short RNAs complementary to the SLA regions were also synthesized with fluorescein: the top stem region of SLA (ssRNA_Top_, 5’F-CCUCAAAGA), the side loop region (ssRNA_Side_, 5’F-AGCUUAGCUC), and modified sequence of ‘Side’ (ssRNA_Side-M_, 5’F-AGCUUAGCUCGCGCGCGC) (Supplementary Fig. 5). The ssRNA_Side-M_ was designed to prevent the homodimer formation of ssRNA_Side_ due to its self-complementary sequence, which would prevent interaction with the side loop of SLA. Extension of the Side RNA with GC repeats (underlined in Side-M sequence) would generate RNA dimer through the (GC)_4_ region, allowing the side loop sequence available for SLA interaction. The tRNA-SLA^DENV^ (1 μM) was incubated with increasing amount of fluorescein-labeled RNAs (1 to 4 μM) in binding buffer (20 mM Tris, pH 7.4, 150 mM NaCl, 2 mM MgCl_2_, and 5% glycerol) for 30 min at room temperature (20 °C). The resulting RNA-RNA complexes were separated by 2% agarose gel in 1x TBE buffer at room temperature. The fluorescein labeled RNAs were analyzed with Amersham Typoon (GE Healthcare, Buckinghamshire, UK) using the LD488 laser for excitation and the Cy2 525BP20 filter.

### Fluorescence-based NS5 and SLA interaction assay

The priming loop deletion mutant of DENV3 NS5 (NS5-Δ6) was constructed by removing 6 amino acids (^795^WSIHAH^800^) in the priming loop^28^. NS5 mutants R22A/K23A, K841A/R842A, K841E/R842E and R856A were generated by site-directed mutagenesis. The wild-type DENV3 NS5 and mutants (NS5-Δ6, R22A/K23A, K841A/R842A, K841E/R842E and R856A) were expressed and purified as previously reported^28^. DENV SLA_80_ was synthesized with 3’-end fluorescein (Midland Certified Reagents, Midland, TX), and interaction between DENV NS5 proteins and SLA_80_ was determined by fluorescent titration using a PC1 spectrofluorometer (ISS, Urbana, IL)^36,47^. Briefly, the direct binding of DENV NS5 to the labeled SLA_80_ was monitored by the fluorescence of fluorescein (λ_ex_ = 480 nm; λ_em_ = 520 nm) in 50 mM Tris-HCl (pH 7.5) containing 100 mM NaCl, 1 mM MgCl_2_, 1 mM DTT, and 10% (w/v) glycerol. The relative fluorescence change is defined as *F*_*i*_/*F*_0_, where *F*_*i*_ is the fluorescence of the nucleic acid solution at a given titration point *i*, and *F*_0_ is the initial fluorescence of the sample. Binding curves were then fit to the following equation using KaleidaGraph software (Synergy Software, PA)^22^. The binding constant, *K*_1_, characterizing the association of SLA with NS5, is defined as

1 $${K}_{1}=\frac{{[{\rm{complex}}]}_{F}}{{[{\rm{SLA}}]}_{F}{[{\rm{NS}}5]}_{F}}$$

where [complex]_F_ is the concentration of the formed complex. The observed fluorescence of the sample at any point of the titration is defined as

2 $${F}_{{obs}}={F}_{F}{[{\rm{SLA}}]}_{F}+{F}_{C}{K}_{1}{[{\rm{SLA}}]}_{F}{[{\rm{NS}}5]}_{F}$$

where *F*_*F*_ and *F*_*C*_ are the molar fluorescence intensities of the free SLA and the formed complex, respectively. Thus, the relative observed change of the SLA fluorescence, Δ*F*_obs_, is then

3 $$\Delta {F}_{{obs}}=\frac{{F}_{{obs}}}{{F}_{F}{[{\rm{SLA}}]}_{T}}=\frac{1}{1+{K}_{1}{[{\rm{NS}}5]}_{F}}+\Delta {F}_{{\max }}\left(\frac{{K}_{1}{[{\rm{NS}}5]}_{F}}{1+{K}_{1}{[{\rm{NS}}5]}_{F}}\right)$$

where Δ*F*_max_ = *F*_*C*_/*F*_*F*_ is the maximum value for the observed relative fluorescence quenching. The assays were performed in triplicate and the mean value was used to calculate the apparent binding constant *K*_*1*_. The binding constant *K*_*1*_ for wild-type NS5 is 5.5 ×10^6^ M^−1^ (K_d_ of 181 nM).

### Construction and viral replication of DENV2 RNA containing SLA mutations

Full-length cDNA of DENV2 (New Guinea C strain) containing SLA mutations were constructed in yeast/*Escherichia coli* shuttle vector as previously described^57,58^. The entire SLA sequence (1-70 nt) was replaced with that of ZIKV in SLA-ZIKV, and the side loop sequence (^44^GAGCUAAGC^52^) was replaced with ^44^AAAAAAAA^52^ in SLA-9A. The DNA fragment containing the desired mutations were generated by overlapping PCR using the primers in Supplementary Table 2. The ZIKV-SLA mutation was generated by three PCR amplifications using the template cDNA pRS424-FLDV2 encoding full-length DENV2 RNA (NGC strain, for 1^st^ and 3^rd^ PCR fragments) and pRS424-FLZIKV (PRVABC-59 strain, for 2nd PCR fragment). The SLA-9A mutant was generated with two PCR amplifications using the pRS424-FLDV2 template (Supplementary Table 2). The final PCR product for SLA-ZIKV or SLA-9A was mixed with the pRS424-FLDV2 cDNA double digested with BsrGI and NgoMIV in competent yeast cell mixture. Both DENV2 SLA recombinant plasmids were created by yeast recombination and selected in TRP(-) media. The DENV2 chimera plasmids (SLA-ZIKV and SLA-9A) were then linearized using the BcgI site at the 3′-end of the viral sequence, and used for in vitro transcription catalyzed by SP6 RNA polymerase (Epicenter Biotechnologies) in the presence of the 7-MeGpppG cap analog.

The DENV2 RNA (∼3 μg) containing either the WT DENV2 SLA, SLA-ZIKV, or SLA-9A was transfected by electroporation (Amaxa Nucleofector II system, Amaxa Biosystems, Cologne, Germany) into BHK-21 cells (American Type Culture Collection, Manassas, VA), as previously described^57^. Briefly, ∼1 × 10^6^ cells were resuspended in 100 μl of Ingenio solution (Mirus Bio, Madison, WI). After pulsing, cells were resuspended in 3 ml of prewarmed complete medium (Dulbecco’s modified Eagle’s medium (DMEM), supplemented with 10% fetal bovine serum and 1× streptomycin/penicillin) in a T-12.5 flask at 37 °C in an CO_2_ incubator. On day 2, cells were trypsinized and transferred into T-75 flask. This procedure was repeated using one-third of the trypsinized cells every 4 days. For immunofluorescence assay, RNA-transfected cells were seeded into a slide (LabTek) and fixed by treatment with acetone. Cells were incubated with a 1:200 dilution of 7E11, a monoclonal antibody against DENV2 NS1. Fluorescein isothiocyante (FITC)-labeled, goat anti-mouse immunoglobulin G conjugate (Kirkegaard & Perry Laboratories) was used as a secondary antibody at a 1:100 dilution. Immunofluorescence photomicrographs (×200 magnification) were acquired using an Olympus IX-71 inverted epifluorescence microscope coupled to the Olympus automated photographic system.

### Sanger DNA sequencing and next-generation sequencing (RNA-seq)

For Sanger sequencing analysis, viral RNA from collected supernatants was amplified by RT-PCR (New England Biolabs) with the viral sequence-specific primers^57^ to make overlapping fragments of ∼800 bp each that spanned the entire genome (Supplementary Table 3). PCR products were purified by agarose gel (Zymo Research), and the nucleotide sequence of each fragment was determined by GENEWIZ Inc.

For next-generation sequencing, viral RNAs were extracted from supernatants (∼10 ml) of BHK-21 cells, as previously described^57^. Briefly, the debris in the supernatants was removed by centrifugation at 355 x g for 5 min, and a 2.5 ml of 40% PEG 8000 was added to 10-ml supernatants. The mixed solutions were stirred at 4 °C for 2 h and refrigerated further for 3 h without stirring. After centrifugation at 15,170 x g for 45 min at 4 °C, each pellet was collected with ∼0.5 ml of the remaining solution and mixed with 1.5 ml of TRIzol-LS (Ambion). Chloroform (0.4 ml) was added and mixed further. The solution was centrifuged at 12,000 x *g* for 15 min, and the upper layer was collected and precipitated by the addition of ethanol. The pellet was resuspended in nuclease-free water, and the final RNA concentrations were prepared in a range between 25 and 100 ng/μl. cDNA library preparation and next-generation sequencing procedures were performed by LC Science (Houston).

After removing ribosomal RNA (rRNA) using NEBNext rRNA depletion kit, RNA samples were treated with the Illumina TruSeq stranded mRNA library prep kit, which consists of RNA fragmentation followed by first- and second-strand cDNA synthesis, adenylation of the 3′-ends, adapter ligation, and DNA fragment enrichment via PCR (10 cycles). The quality control check of the libraries using an Agilent 2100 Bioanalyzer showed a range of library sizes, with the curve being mostly between 200 and 400 base pairs long. The libraries were pooled together with equal amounts based on the Agilent quantification. Real-time qPCR was used to quantify the pooled libraries for optimal clustering during sequencing. All samples were sequenced on a single Illumina Novaseq 6000 run, generating paired-end 150-base-pair readings. The .bcl files generated by the sequencer were converted to Fastq data using the software bcl2fastq v2.17.1.14. DENV2 genomes were aligned with Geneious prime software (Biomatters), which differentiates statistically significant variants from total SNPs identified within reads. Briefly, paired-end sequences were made from Fastq data R1 and R2. Sequenced fragments (contigs) were trimmed with the quality scores limit set to 0.05. The trimmed contigs were aligned to the reference (input viral cDNA) sequence, and the SNPs were analyzed. Nucleotide alterations at more than 3% frequency are shown in Fig. 4c.

### Reporting summary

Further information on research design is available in the Nature Research Reporting Summary linked to this article.

## Supplementary information

Supplementary Information

Reporting Summary

## Data Availability

The data that support this study are available from the corresponding authors upon reasonable request. The atomic coordinates and structure factors have been deposited in the Protein Data Bank under the accession codes 7LYF and 7LYG for tRNA-SLA^DENV^ and tRNA-SLA^ZIKV^, respectively. The RNA-seq data as raw reads are available as fastq files with accession code PRJNA662929 at NCBI Short Read Archive (SRA) database. Source data are provided with this paper.

## Source data

Source Data
