# Supplementary material for: Structures of flavivirus RNA promoters suggest two binding modes with NS5 polymerase
Source: Nat Commun. 2021 May 5;12:2530. doi: 10.1038/s41467-021-22846-1 (PMC8100141; doi:10.1038/s41467-021-22846-1)
Supplement: Supplementary file 1 — Supplementary Information [file 41467_2021_22846_MOESM1_ESM.pdf]

Structures of flavivirus RNA promoters suggest two binding modes with NS5 polymerase

Eunhye Lee[1], Paul J. Bujalowski[1], Tadahisa Teramoto[2], Keerthi Gottipati[1], Seth D. Scott[1], Radhakrishnan Padmanabhan[2], and Kyung H. Choi[1,*]

[1] Department of Biochemistry and Molecular Biology, Sealy Center for Structural Biology and Molecular Biophysics, The University of Texas Medical Branch, 301 University Boulevard, Galveston, TX 77555, USA

[2] Department of Microbiology and Immunology, Georgetown University School of Medicine, Washington, D.C. 20057, USA.

Supplementary Information

Supplementary Methods

Supplementary Fig 1 – 7

Supplementary Table 1 - 3

Supplementary Methods

Fluorescence-based NS5 and SLA interaction assay

Fluorescence-based competition assay was used to measure binding affinity between DENV NS5 and tRNA-SLA^{DENV}, as previously described [22]. Briefly, binding of NS5 and etheno-derivative of A(pA)$_{19}$, εA(pεA)$_{19}$ was carried out in buffer A (150 mM NaCl, 50 mM Tris, pH 7.5, 2mM 2-mercaptoethanol and 10% glycerol) at 10 °C in the absence and presence of tRNA-SLA^{DENV}, and the changes of the fluorescence signal originating from εA(pεA)$_{19}$ were monitored (λ_{ex} = 325 nm; λ_{em} = 410 nm). The concentrations of εA(pεA)$_{19}$ and tRNA-SLA were 8.0×10^{-7} M and 3.2×10^{-6} M, respectively. Binding curves were fit to the equations 1 and 2 using KaleidaGraph software (Synergy Software, PA) and the binding constants K_1 and K_2, describing the association of εA(pεA)$_{19}$ and tRNA-SLA^{DENV} with NS5, respectively, were determined.

$$\Delta F_{obs} = \frac{F_{obs}}{F_F[F_{RNA}]_T} = \frac{1}{1 + K_1[NS5]_F} + \Delta F_{max}\left(\frac{K_1[NS5]_F}{1 + K_1[NS5]_F}\right) \qquad (1)$$

$$[NS5]_{total} = [NS5]_F + \frac{K_1[F_{RNA}]_T[NS5]_F}{1 + K_1[NS5]_F} + \left(\frac{K_2[SLA]_T[NS5]_F}{1 + K_1[NS5]_F}\right) \qquad (2)$$

where $\Delta F_{max} = F_C/F_F$, is the maximum value of the observed relative fluorescence quenching, and F_F and F_C are the molar fluorescence intensities of the free εA(pεA)$_{19}$ and the formed complex, respectively.

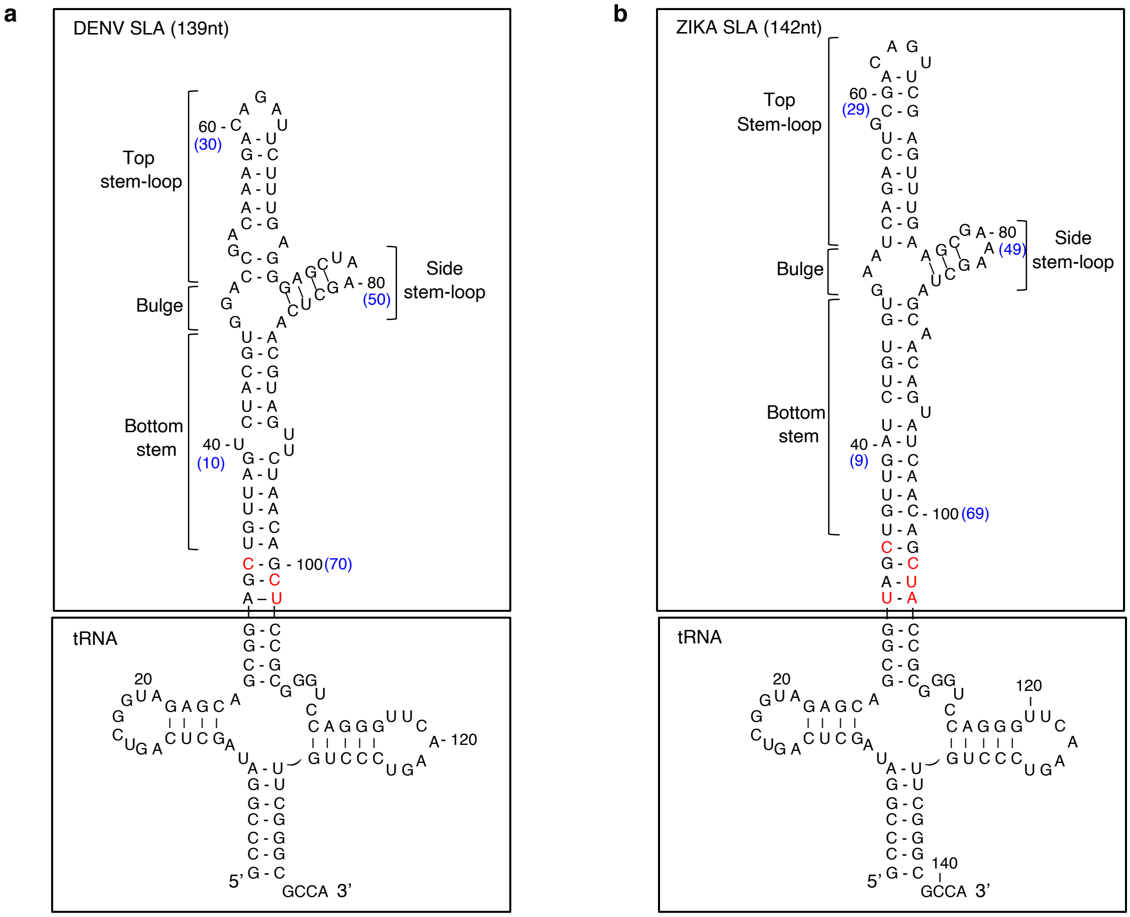

Supplementary Fig. 1 Design of chimeric tRNA-SLAs. a, The predicted secondary structure of tRNA-SLA^{DENV}. DENV2 SLA (nucleotides 1-70 of NC_001474) is inserted into the anticodon loop between the 5'- and 3'- human tRNA^{Lys} scaffold sequence to create tRNA-SLA^{DENV}. To stabilize the chimeric RNA, two modifications were introduced; ^{33}U to C mutation and ^{101}CU^{102} insertion (indicated in red). The mutations were introduced to form a continuous dsRNA helix in the linker region between tRNA^{Lys} scaffold and SLA. The predicted secondary structure of the chimeric construct by RNAfold server [51] indicates that the RNA chimera maintains the same predicted folds for individual tRNA and SLA. The nucleotide numbers in SLA are also shown in parenthesis in blue. **b**, The secondary structure of tRNA-SLA^{ZIKV}. The SLA sequence of epidemic strain ZIKV (nucleotides 1-71 of KU527068) was inserted into the anticodon loop between the 5'- and 3'- human tRNA^{Lys} scaffold sequence. The chimeric construct was further modified to stabilize the structure and to get better diffracting crystals. The final construct contains the ^{34}U to C substitution, ^{103}CU^{104} insertion, and one base pair (^{31}U-^{105}A) insertion in the linker region between tRNA^{Lys} scaffold and SLA (indicated in red).

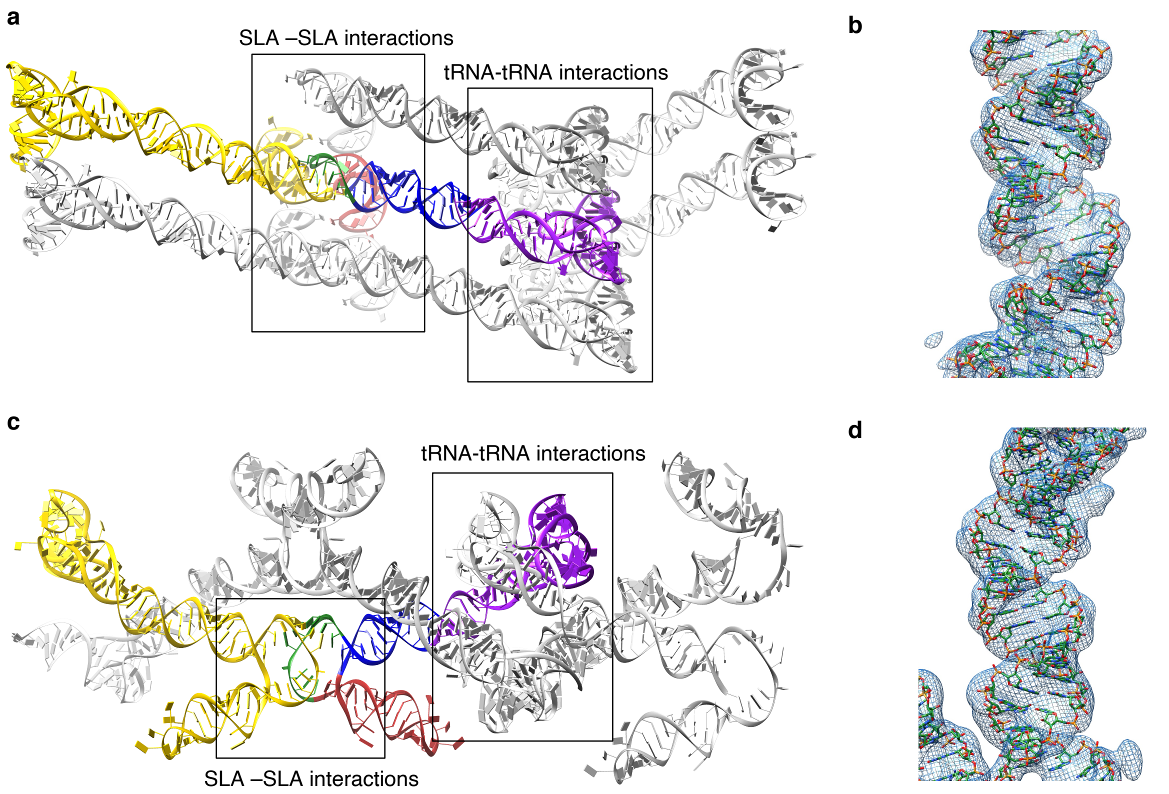

Supplementary Fig. 2 Crystal packing of tRNA-SLA^{DENV} and tRNA-SLA^{ZIKV}. **a, c,** Crystal packing environment is shown with symmetry related molecules for tRNA-SLA^{DENV} (**a**) and tRNA-SLA^{ZIKV} (**c**). Asymmetric unit contains single tRNA-SLA in both structures. One tRNA-SLA molecule is colored by the RNA regions (red, top stem-loop; green, side loop; blue, bottom stem; purple, tRNA), and the neighboring molecule that forms an inter-molecular three-way junction is shown in yellow. All other molecules are shown in grey. The crystal packing is mediated by SLA-SLA and tRNA-tRNA interactions. **b, d,** A section of a composite omit map contoured at 1.2 σ for tRNA-SLA^{DENV} (**b**) and tRNA-SLA^{ZIKV} (**d**).

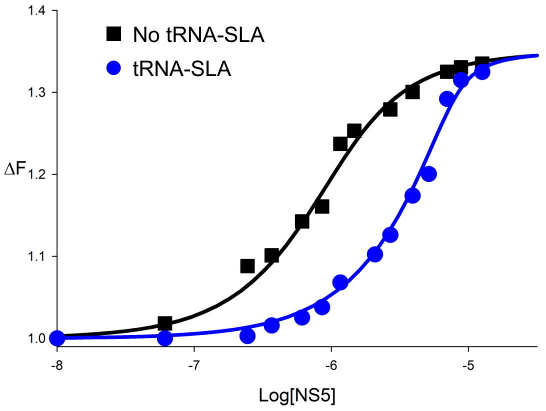

Supplementary Fig. 3. Binding of DENV NS5 with tRNA-SLA^{DENV}. Fluorescence titrations of fluorescent etheno-adenosine 20mer, $\varepsilon A(p\varepsilon A)_{19}$ with DENV3 NS5 (λ_{ex} = 325 nm; λ_{em} = 410 nm) were carried out in the buffer A (150 mM NaCl, 50 mM Tris, pH 7.5, 2 mM 2-mercaptoethanol and 10% glycerol) at 10°C in the absence and presence of tRNA-SLA^{DENV}. The concentration of $\varepsilon A(p\varepsilon A)_{19}$ is 8.0×10^{-7} M and tRNA-SLA is 3.2×10^{-6} M. When tRNA-SLA^{DENV} is present, the titration curve shifted toward higher NS5 concentrations, indicating competition between $\varepsilon A(p\varepsilon A)_{19}$ and tRNA-SLA for NS5. The solid lines are nonlinear least-squares fits of the titration curves with $K_{F\text{-}RNA} = 2.1 \times 10^6$ M^{-1} and $K_{tRNA\text{-}SLA} = 4.8 \times 10^6$ M^{-1}.

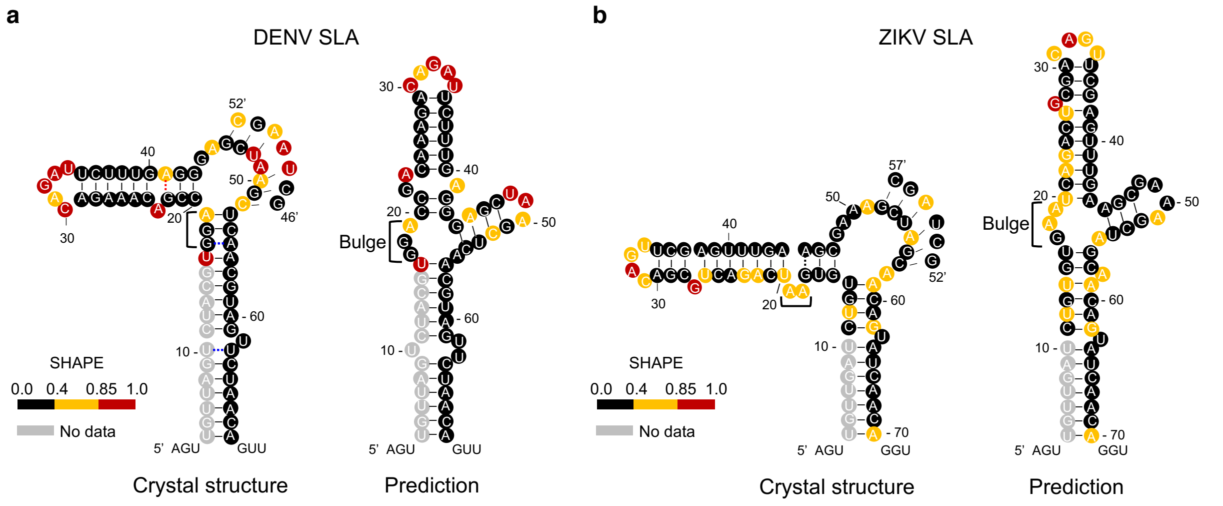

Supplementary Fig. 4 Comparison of the SLA structures with SHAPE data. **a,** SHAPE reactivity of DENV SLA. The normalized SHAPE reactivities determined for DENV SLA[30] are mapped on the secondary structures of crystal structure or the RNAfold prediction. The bulge in the predicted structure and the corresponding region in the crystal structure are indicated by brackets. The side loop sequence (nt 46 to 52) of a neighboring molecule that form kissing loop interactions are indicated. **b,** SHAPE reactivity of ZIKV SLA. The normalized SHAPE reactivities determined for ZIKV SLA[29] are mapped on the secondary structures of crystal structure or the RNAfold prediction. The bulge in the predicted structure and the corresponding region in the crystal structure are indicated by brackets. The side loop sequence (nt 52 to 57) of a neighboring molecule that form kissing loop interactions are shown. In both DENV and ZIKV SLAs, the bulge region does not show high SHAPE reactivity expected from the secondary structure predictions.

Fluorescein-labeled RNAs		
**SLA**		**SLA(-)**
5 '- AGUUGUUGAUCUGUGUGAAUCAGACUGCGACAG UUCGAGUUUGAAGCGAAAGCUAGCAACAGUAUCAAC AGGUUUUAUUUU-3' 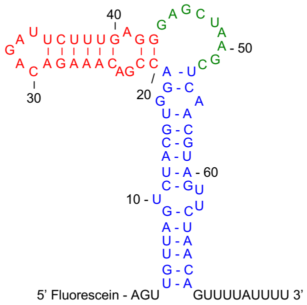		5' - AACUGUUAGAACUACGUUGAGCUUAGCUCCCU CAAAGAAUCUGUCUUUGUCGGUCCACGUAGACUAA CAACU - 3' 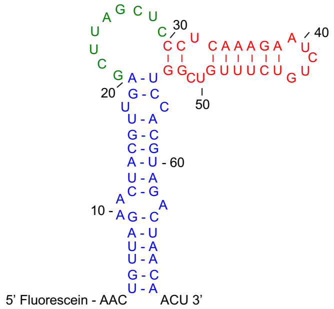
**8-mer**	5' - AGAAAAGG - 3'	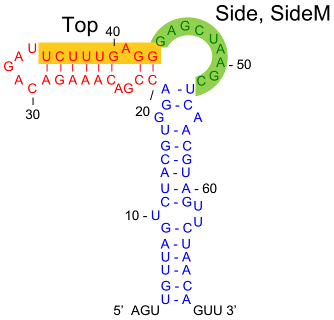
**Top**	5' - CCUCAAAGA - 3'	
**Side**	5' - AGCUUAGCUC - 3'	
**SideM**	5' - AGCUUAGCUCGCGCGCGC - 3'	

Supplementary Fig. 5 Fluorescein-labeled RNAs used in RNA-RNA interaction assay with tRNA-SLA^{DENV}. Fluorescein-labeled RNAs used in Fig. 4b are shown along with the secondary structures of SLA and SLA(-), and the target binding sites for Top and Side sequences. SLA, SLA in the 5'-end of positive strand; SLA(-), the complementary sequence of SLA in the 3'-end of negative strand; 8-mer, random 8-mer RNA; Top, complementary sequence of top stem (nt 35-43); Side, complementary sequence of the side loop (nt 44-52) with addition of A base at the 5' end; Side-M, complementary sequence of the side loop (nt 44-52) with $(GC)_4$ extension at the 3' end. Note that the Side and Side-M can form homodimers using self-complementary sequences.

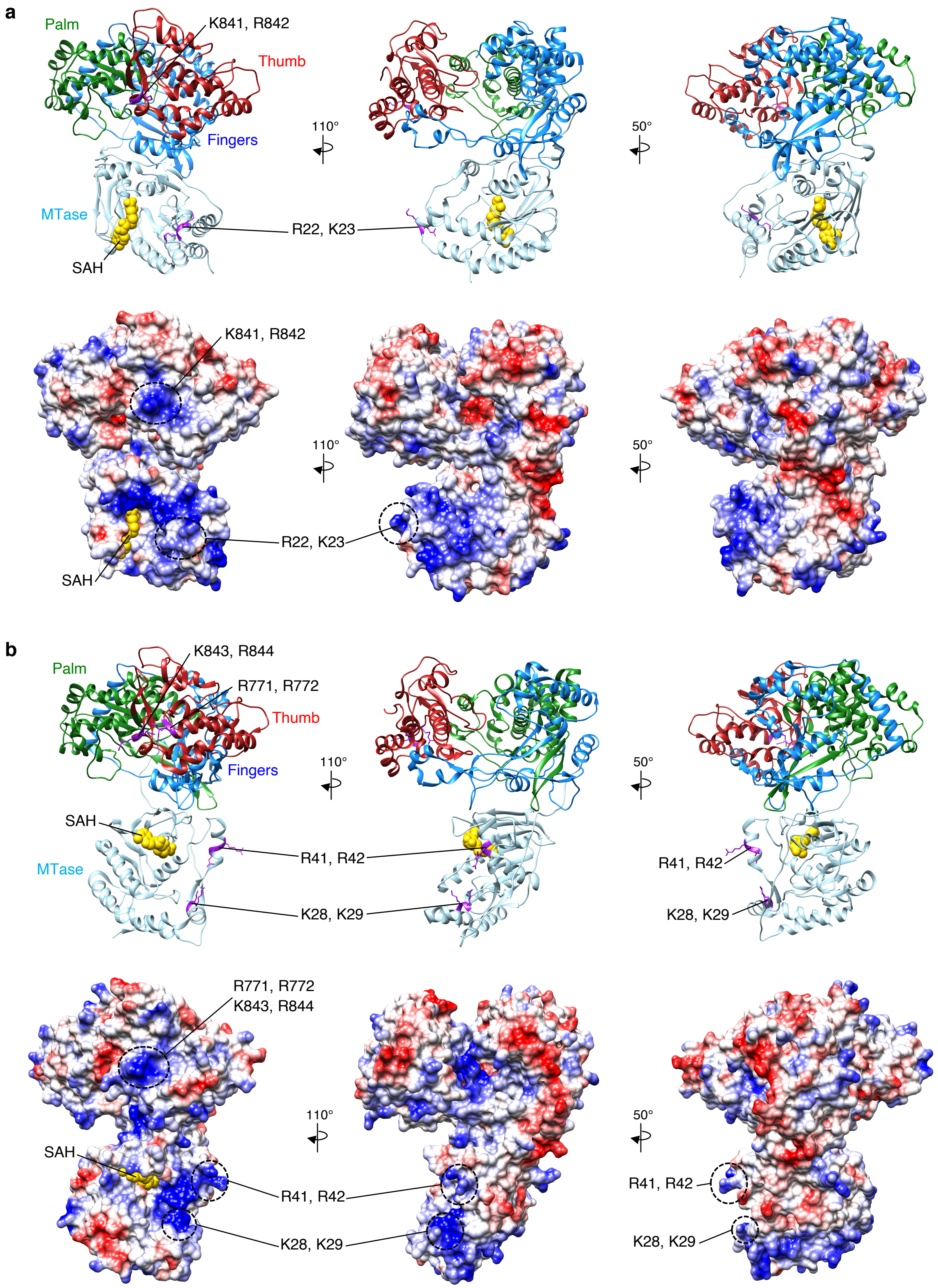

a
Palm
K841, R842
Thumb
Fingers
MTase
SAH
110°
50°
R22, K23
K841, R842
110°
50°
R22, K23
SAH
b
Palm
K843, R844
R771, R772
Thumb
Fingers
110°
50°
SAH
MTase
R41, R42
K28, K29
R41, R42
K28, K29
R771, R772
K843, R844
110°
50°
SAH
R41, R42
K28, K29
R41, R42
K28, K29

Supplementary Fig. 6 Proposed SLA-binding site in DENV and ZIKV NS5. a, The SLA-binding site in DENV NS5. DENV NS5 (PDB code 4V0Q) is composed of two domains, methyltransferase (MTase, cyan) and RNA-dependent RNA polymerase (RdRp). The RdRp domain is composed of the fingers (blue), palm (green), and thumb (red) regions. SAH, a byproduct of the MTase reaction, bound in the MTase active site is shown as yellow spheres. The NS5 structure is shown in three views along with their electrostatic surface representations (bottom). The residues identified as the SLA-binding site in the DENV NS5-SLA binding assay (R22, K23, K841, and R842) are indicated. **b**, The SLA-binding site in ZIKV NS5. ZIKV NS5 (PDB code 5U0B) is shown in three views with their electrostatic surface representations. The residues identified as the SLA-binding site in the ZIKV NS5-SLA binding assay (K28, R29, R41, R42, R771, R772, K843, and R844) are indicated [46].

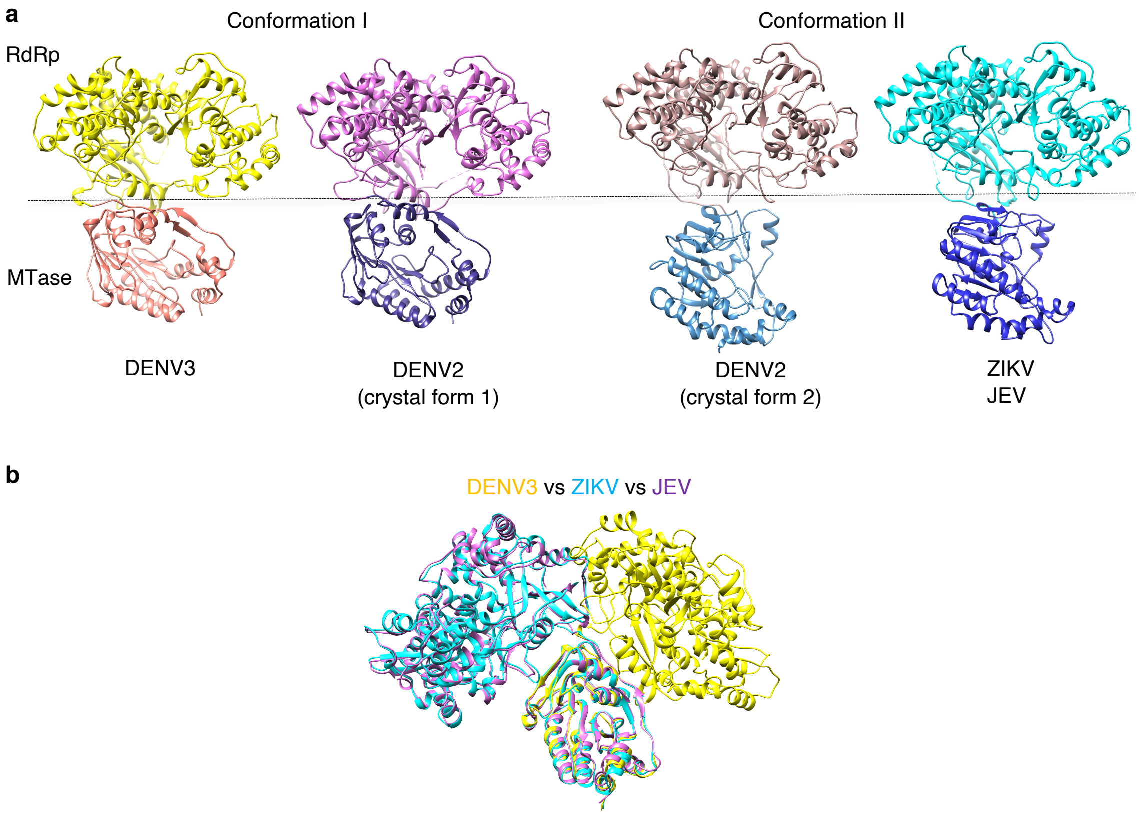

Supplementary Fig. 7 Flavivirus NS5 structures show two domain orientations. a, The NS5 structures of flaviviruses. NS5 structures of DENV serotype 3 (PDB ID: 4V0Q), two crystal forms of DENV serotype 2 (PDB IDs: 6KR3 and 6KR2), JEV (PDB ID: 4K6M), and ZIKV (PDB ID: 5U0B) are shown in ribbon model. **b**, Superposition of the NS5 structures of DENV3, JEV, and ZIKV. When the MTase domains are superposed, the RdRp domains show two distinct orientations. The NS5 structure of DENV3 (yellow) show a one orientation and ZIKV (cyan) and JEV (pink) show the other.

Supplementary Table 1. X-ray crystallographic data collection and refinement statistics

	tRNA-SLA^{DENV}	tRNA-SLA^{ZIKV}
Data collection		
Wavelength (Å)	0.97985	1.12713
No. of reflections	10,308	6864
Space group	I222	C222
Unit cell dimensions (Å)	a=30.92, b=137.56, c=336.88	a=157.07, b=156.58, c=72.32
Resolution	50-3.4 (3.46-3.37)*	50-3.8 (3.89-3.80)
Completeness (%)	97.2 (72.3)	95.9 (81.7)
Redundancy	7.1 (3.6)	10.4 (8.3)
I/σI	37.0 (1.1)	23.4 (0.8)
Rp.i.m	0.028 (0.480)	0.025 (0.435)
CC1/2 (highest resolution shell)	0.991 (0.636)	0.998 (0.661)
CC* (highest resolution shell)	0.998 (0.882)	0.999 (0.892)
Refinement		
Resolution range (Å)	50 - 3.4	50 - 3.8
Rwork/Rfree	0.226/0.268	0.227/0.272
No. of atoms		
Nucleotide	2972	3039
Ligand/ion	1 Mg, 7 waters	0
r.m.s deviations		
Bond length (Å)	0.001	0.003
Bond angles (°)	1.281	0.678
B-factors (Å^2)		
Nucleotide	155	224
Ligand/ion	129	

* values in parentheses are for the highest resolution shell.

Supplementary Table 2. PCR primers used to generate SLA mutants

SLA-ZIKV	1st PCR<br>forward: 5' TCGAGGTGCCGTAAAGCACTA 3'<br>reverse: 5' GATTCACACAGATCAACAACTCTATAGTGTCACCTAAATTC 3'
	2nd PCR<br>forward: 5' GAATTTAGGTGACACTATAGAGTTGTTGATCTGTGTGAATC 3'<br>reverse: 5' GATCTGCTCTCTAATTAAAAACTGTTGATACTGTTGCTAGC 3'
	3rd PCR<br>forward: 5' AGCTAGCAACAGTATCAACAGTTTTTAATTAGAGAGCAGATC 3'<br>reverse: 5' CCTTCCAATCTCTTTCCTGAA 3'
SLA-9A	1st PCR<br>forward: 5' TCGAGGTGCCGTAAAGCACTA 3'<br>reverse: 5' GTTGATTTTTTTTTCCTCAAAGAATCTGTCTTTGTC 3'
	2nd PCR<br>forward: 5' TGAGGAAAAAAAAATCAACGTAGTTCTAACAGTTTTT 3'<br>reverse: 5' CCTTCCAATCTCTTTCCTGAA 3'

Supplementary Table 3. Primers used for Sanger sequencing of the cDNAs of viral RNAs

Primer number [a]	Primer sequence [b]
9F<br>846R	TCT ACG TGG ACC GAC AAA GAC<br>CAT TAT GGT AAA GCC TGG ATG
255F<br>1100R	GTG GCG TTC CTT CGT TTC CTA<br>AGT GGC AGG AGG TTG TTT GGC TTC
962F<br>1450R	AGA GAC TTT GTA GAA GGG GTT<br>TGT GAT GGA ACT CTG TGG TGT
1310F<br>2300R	GTC GTG CAA CCA GAA AAC TTG<br>GAC TCC TAT GAG GAT TTT CAT
1900F<br>2644R	ATC AGA GTA CAA TAT GAA GGG<br>ATT CTG GTG TTA TTTGTT TCC
2500F<br>3260R	ACA TGG ACA GAA CAA TAC AAG<br>CAC CAC TGT GGT TCC TTC GCA
2970F<br>3700R	CAT GTC AGC GGC CAT AAA AGA<br>ATA AGT CAC GCC CAT ACC TAT
3605F<br>4460R	TGA TCA CAG GGA ACA TGT CCT<br>AAA AAG TCC TGA GAT CAC CAG
4329F<br>5160R	AGA TAT CAG GAA GCA GTC CAA<br>TTT ATA GCC TCT CTG ACT ATG
5100F<br>5700R	CAC CCA GGA GCG GGA AAG ACG<br>GGT TCT AGT CTT GAC ATA CTC
5600F<br>6400R	GTT CGT TCC AAG TAT AAA AGC<br>TGT GAT TAG GTT CAG GGT CAG
6000F<br>6700R	CTC CTA GAT AAC ATC AAC ACA<br>TAT TGA AGC TGC TAT CCA GTG
6600F<br>7300R	GAA GGG GTA TAG GGA AGA TGA<br>TAT TGG ATC TAG GTC AAT CAC
7000F<br>7700R	CTC AGT GAA CGT GTC CCT AAC<br>GCC TTC TTT TGC TAA GGT TCT
7607F<br>8600R	GGA AAA GCC GAT TGA ACG CAT TG<br>TGT CAT TGC CAT CTG TGT CAC
8500F<br>9300R	AAC AAA CTG GAT CAG CAT C<br>CAC ACG CAC CAC CTT GTT TTG
9220F<br>9948R	AAT GGT AAC AAA CCA CAT GG<br>TGT TCT ACT TGT TGG AAC CCA
9817F<br>10520R	GAG ACG GCC TGT TTG GGG AAG<br>AAG GGA GGG GTC TCC TCT AAC
10455F<br>3'end	TCT GGG AGG CCA CAA ACC ATG<br>AGA ACC TGT TGA TTC AAC AGC

[a] Oligonucleotide primers for PCR reactions are described as pairs. The numbers refer to the positions in the DENV2 genome (New Guinea C-strain). F and R refer to forward and reverse primers, respectively.

[b] The nucleotide sequences are described as 5' to 3' direction.
