# Supplementary material for: Structures of flavivirus RNA promoters suggest two binding modes with NS5 polymerase
Source: Nat Commun. 2021 May 5;12:2530. doi: 10.1038/s41467-021-22846-1 (PMC8100141; doi:10.1038/s41467-021-22846-1)
Supplement: Supplementary file 2 — Reporting Summary [file 41467_2021_22846_MOESM2_ESM.pdf]

nature research

Corresponding author(s): Radhakrishnan Padmanabhan and Kyung H. Choi

Last updated by author(s): Mar 19, 2021

Reporting Summary

Nature Research wishes to improve the reproducibility of the work that we publish. This form provides structure for consistency and transparency in reporting. For further information on Nature Research policies, see our Editorial Policies and the Editorial Policy Checklist.

Statistics

For all statistical analyses, confirm that the following items are present in the figure legend, table legend, main text, or Methods section.

n/a	Confirmed	
[ ]	[x]	The exact sample size (*n*) for each experimental group/condition, given as a discrete number and unit of measurement
[ ]	[x]	A statement on whether measurements were taken from distinct samples or whether the same sample was measured repeatedly
[x]	[ ]	The statistical test(s) used AND whether they are one- or two-sided<br>*Only common tests should be described solely by name; describe more complex techniques in the Methods section.*
[x]	[ ]	A description of all covariates tested
[x]	[ ]	A description of any assumptions or corrections, such as tests of normality and adjustment for multiple comparisons
[ ]	[x]	A full description of the statistical parameters including central tendency (e.g. means) or other basic estimates (e.g. regression coefficient) AND variation (e.g. standard deviation) or associated estimates of uncertainty (e.g. confidence intervals)
[x]	[ ]	For null hypothesis testing, the test statistic (e.g. *F*, *t*, *r*) with confidence intervals, effect sizes, degrees of freedom and *P* value noted<br>*Give P values as exact values whenever suitable.*
[x]	[ ]	For Bayesian analysis, information on the choice of priors and Markov chain Monte Carlo settings
[x]	[ ]	For hierarchical and complex designs, identification of the appropriate level for tests and full reporting of outcomes
[x]	[ ]	Estimates of effect sizes (e.g. Cohen's *d*, Pearson's *r*), indicating how they were calculated

Our web collection on statistics for biologists contains articles on many of the points above.

Software and code

Policy information about availability of computer code

Data collection	X-ray data were collected at the Advanced Photon Source beamline 21-ID-F using the a web-based remote access.
Data analysis	X-ray data were processed with HKL2000; structures were solved and refined with the PHENIX suite 1.14. The binding assay results were analyzed with KaleidaGraph version 4.03.

For manuscripts utilizing custom algorithms or software that are central to the research but not yet described in published literature, software must be made available to editors and reviewers. We strongly encourage code deposition in a community repository (e.g. GitHub). See the Nature Research guidelines for submitting code & software for further information.

Data

Policy information about availability of data

All manuscripts must include a data availability statement. This statement should provide the following information, where applicable:

- Accession codes, unique identifiers, or web links for publicly available datasets
- A list of figures that have associated raw data
- A description of any restrictions on data availability

The atomic coordinates and structure factors have been deposited in the Protein Data Bank under the accession codes 7LYF (https://www.rcsb.org/structure/unreleased/7LYF) and 7LYG (https://www.rcsb.org/structure/unreleased/7LYG) for tRNA-SLADENV and tRNA-SLAZIKV, respectively. The RNAseq data as raw reads are available as fastq files (accession code PRJNA662929) at NCBI Short Read Archive (SRA) database (https://www.ncbi.nlm.nih.gov/sra/?term=PRJNA662929).

Field-specific reporting

Please select the one below that is the best fit for your research. If you are not sure, read the appropriate sections before making your selection.

[x] Life sciences   [ ] Behavioural & social sciences   [ ] Ecological, evolutionary & environmental sciences

For a reference copy of the document with all sections, see nature.com/documents/nr-reporting-summary-flat.pdf

Life sciences study design

All studies must disclose on these points even when the disclosure is negative.

Sample size	The binding assays were performed in triplicate (n=3 independent experiments).
Data exclusions	No data were excluded from the analyses.
Replication	The binding assays were performed in triplicate, and all attempts were successful.
Randomization	N/A. The binding assays were done with increasing amount of proteins. Randomization is not applicable in this type of experiments.
Blinding	N/A. The fluorescence-based binding assay requires the knowledge of exact concentrations of RNA and protein.

Reporting for specific materials, systems and methods

We require information from authors about some types of materials, experimental systems and methods used in many studies. Here, indicate whether each material, system or method listed is relevant to your study. If you are not sure if a list item applies to your research, read the appropriate section before selecting a response.

Materials & experimental systems

n/a	Involved in the study
[ ]	[x] Antibodies
[ ]	[x] Eukaryotic cell lines
[x]	[ ] Palaeontology and archaeology
[x]	[ ] Animals and other organisms
[x]	[ ] Human research participants
[x]	[ ] Clinical data
[x]	[ ] Dual use research of concern

Methods

n/a	Involved in the study
[x]	[ ] ChIP-seq
[x]	[ ] Flow cytometry
[x]	[ ] MRI-based neuroimaging

Antibodies

Antibodies used	Anti-NS1 monoclonal antibody, 7E11, was obtained from Dr. Robert Putnak at Walter Reed Army Institute of Research. The antibody is described by Henchal EA et al., (Rapid identification of dengue virus isolates by using monoclonal antibodies in an indirect immunofluorescence assay, Am J Trop Med Hyg. 1983 32(1):164-9). FITC-labeled goat polyclonal anti-mouse immunoglobulin G (H +L) antibody is available from Seracare, Inc. (material number 5230-0307, lot number 10319966).
Validation	This Mab has been validated in numerous independent experiments which have been already published.

Eukaryotic cell lines

Policy information about cell lines

Cell line source(s)	BHK-21 cell line was purchased from ATCC. The cell line was expanded immediately and divided into aliquots for frozen vials kept at liquid nitrogen tank.
Authentication	authentication from ATCC
Mycoplasma contamination	TFor each experiment, fresh culture was started from a frozen vial and the lack of mycoplasma contamination was confirmed by PCR at the beginning and the end of the experiment. PCR test for mycoplasma contamination has been performed with Uphoff and Drexler method (Comparative PCR analysis for detection of mycoplasma infections in continuous cell lines. Uphoff CC, Drexler HG.In Vitro Cell Dev Biol Anim. 2002 Feb;38(2):79-85)
Commonly misidentified lines (See ICLAC register)	None
